# The first CT-based classification system for pulmonary actinomycosis: correlating imaging patterns with therapeutic strategies and prognosis in a highly misdiagnosed disease

**DOI:** 10.3389/fcimb.2026.1836090

**Published:** 2026-07-02

**Authors:** Guangzhao Zhang, Tianyu Yao, Wuying Yuan, Yanfei He, Kunying Li

**Affiliations:** 1 Department of Thoracic Surgery/Minimally Invasive Surgery, Henan Provincial Chest Hospital/Affiliated Chest Hospital of Zhengzhou University, Henan Branch of National Regional Medical Center for Infectious Disease, Zhengzhou, Henan, China; 2 Department of Medical Imaging, Henan Provincial Chest Hospital/Affiliated Chest Hospital of Zhengzhou University, Henan Branch of National Regional Medical Center for Infectious Disease, Zhengzhou, Henan, China; 3 Endoscopic Diagnosis and Treatment Center. Henan Provincial Chest Hospital/Affiliated Chest Hospital of Zhengzhou University, Henan Branch of National Regional Medical Center for Infectious Diseases, Zhengzhou, Henan, China

**Keywords:** classification, computed tomography, differential diagnosis, misdiagnosis, prognosis, pulmonary actinomycosis

## Abstract

**Background:**

Pulmonary actinomycosis is a rare, chronic, suppurative infection caused by anaerobic, Gram-positive Actinomycesspecies, characterized by a notoriously high misdiagnosis rate. Due to non-specific clinical and imaging presentations, it is frequently misdiagnosed. This study aims to establish the first CT-based classification system to improve diagnostic accuracy and guide individualized treatment.

**Methods:**

A retrospective analysis was conducted on patients with pathologically or molecularly confirmed pulmonary actinomycosis between February 2003 and April 2024. Their chest CT features were systematically analyzed to establish the classification.

**Results:**

Sixty-nine patients were enrolled (mean age 53.3 ± 12.1 years; 57 males). Based on chest CT features, the disease was categorized into five types: Type I: Nodular infiltrative (8.7%); Type II: Airspace suspension (73.9%), the most prevalent; Type III: Bilateral disseminated (2.9%), relatively rare; Type IV: Airway-originated (8.7%); Type V: Occult manifestation (5.8%). Although the postoperative complication rate was 50.0%, patients achieved a high cure rate following surgical resection combined with long-term antibiotics. The Occult manifestation type responded favorably to conservative treatment. The overall cure rate was 87.0% (60/69), with cure rates of 90.0% in surgical patients and 82.8% in non-surgical patients.

**Conclusion:**

This study introduces the first CT-based classification system for pulmonary actinomycosis, which improves preoperative diagnostic accuracy and guides therapeutic decision-making. The classification types are strongly associated with distinct treatment strategies and prognosis. Applying this system could reduce misdiagnosis and inform optimal management. Future validation in multicenter cohorts is essential to refine and generalize this framework.

## Introduction

Pulmonary actinomycosis is a rare, chronic suppurative infection caused by anaerobic, Gram-positive Actinomyces species, characterized histopathologically by localized suppurative lesions and fibrogranulomatous inflammation. Based on current data, the annual incidence of pulmonary actinomycosis ranges from 1/30, 000 to 1/300, 000. Despite its low incidence, this disease may account for up to 15% of fatal pulmonary conditions (Boot et al., 2023). Despite its relatively low incidence, the non-specific clinical and radiological features of this disease frequently lead to misdiagnosis as pulmonary tuberculosis, lung cancer, lung abscess, fungal pneumonia, or nocardiosis (Mabeza and Macfarlane, 2003; Kassab et al., 2016; Grzywa-Celińska et al., 2017; Ferreira et al., 2023). Studies indicate that pulmonary actinomycosis carries a misdiagnosis rate exceeding 80%, resulting in delayed treatment and increased risks of unnecessary surgical interventions and postoperative complications (Baik et al., 1999; Baykal et al., 2022; Yi et al., 2024). For instance, Martínez-Girón et al. noted that actinomycotic lesions often extend beyond imaging-assessed boundaries, with intraoperative findings revealing more extensive involvement than suggested by preoperative imaging, further complicating clinical management (Martínez-Girón and Pantanowitz, 2021).

Currently, the diagnosis of pulmonary actinomycosis primarily relies on histopathological biopsies (e.g.,bronchoscopic biopsy, CT-guided percutaneous biopsy, or surgical specimens) and microbiological identification (Boot et al., 2023). However, due to the insidious onset and non-specific clinical manifestations, preoperative diagnostic accuracy remains suboptimal, leading to suboptimal therapeutic strategies. Recent advancements in imaging technology have highlighted the value of chest computed tomography (CT) in diagnosing pulmonary diseases. Nevertheless, existing studies predominantly focus on imaging characteristics of isolated cases, differential diagnoses, or descriptive analyses (Ge et al., 2019; Zhang et al., 2020; Baykal et al., 2022; Zhang et al., 2024), and no systematic classification system for chest CT manifestations has been established. Although prior literature has documented typical CT features—such as chronic segmental atelectasis, airspace formation, localized consolidation, low-attenuation necrotic areas, and peripheral enhancement (Kim et al., 2006)—a standardized classification framework that systematically correlates imaging morphology with treatment decisions (surgical versus conservative) and patient outcomes (complications, cure rate) remains absent. This study represents the first attempt to fill this gap.

Furthermore, treatment strategies for pulmonary actinomycosis—including antibiotic selection, surgical resection extent, and postoperative complication management—largely depend on the anatomical location, extent, and invasiveness of the lesions (Guglielmi et al., 1991; Mabeza and Macfarlane, 2003; Ito, 2021; Boot et al., 2023). For example, lesions involving multiple lobes and/or segments, or those involving the pleura, are prone to postoperative complications such as bronchopleural fistula and surgical site infection, whereas localized lesions can result in favorable outcomes through parenchyma-preserving resections (such as segmentectomy or wedge resection) (Rizzi et al., 1996). Nevertheless, the lack of an imaging-based classification system to guide individualized therapeutic decisions remains a significant clinical challenge.

Given the current diagnostic and therapeutic landscape of this disease, the present study aims to:

Systematically describe, summarize, and analyze the characteristics of chest CT manifestations in pulmonary actinomycosis, delineating key differentiating features from other pulmonary pathologies such as tuberculosis and lung cancer;

Establish a CT-based classification system and analyze its correlation with treatment selection and postoperative complication rates;

Investigate the clinical utility of this imaging classification in reducing misdiagnosis rates and optimizing therapeutic strategies;

Establish a CT imaging-based decision-making framework for radiologists and clinicians, facilitating evidence-guided selection of biopsy modalities and therapeutic approaches.

Collectively, this research may provide novel insights into early diagnosis, precision treatment, and prognostic management of pulmonary actinomycosis, while establishing a basis for future multicenter studies and guideline development.

## Methods

### Study design and patients

This retrospective case series study, conducted at Henan Provincial Chest Hospital, enrolled patients with pathologically confirmed pulmonary actinomycosis between February 2003 and April 2024. All participants had complete clinical records and chest CT imaging archives. Inclusion Criteria: 1) Definitive diagnosis via histopathological biopsy (bronchoscopic biopsy, CT-guided percutaneous biopsy, or surgical specimen) or metagenomic next-generation sequencing (mNGS) or microbiological identification, molecular diagnostic techniques (e.g., 16S rRNA PCR); 2) Clinical manifestations consistent with pulmonary actinomycosis (e. g., chronic suppurative lesions, fistula formation, chest pain, hemoptysis), supported by chest CT findings and laboratory tests; 3) Availability of preoperative or diagnostic-phase chest CT scans (including non-contrast and contrast-enhanced studies) with sufficient image quality to visualize key lesion characteristics (e. g., hypodense necrotic areas, peripheral enhancement, *Airspace suspension Sign*); 4) Diagnosis confirmed within the study period (February 2003–April 2024 at Henan Provincial Chest Hospital);5) Age ≥18 years. Exclusion Criteria: 1) Suspected cases lacking histopathological/microbiological confirmation; absence of preoperative/diagnostic CT scans or scans with suboptimal quality (e. g., significant artifacts, motion blur) precluding accurate lesion assessment; 2) Concurrent severe pulmonary diseases potentially altering CT features (e.g. diffuse pulmonary fibrosis, emphysema) or systemic conditions (e. g., immunosuppression); 3) Prior high-dose antibiotic therapy or interventions significantly modifying CT imaging findings before diagnosis. This study was approved by the Ethics Committee of Henan Provincial Chest Hospital (Approval No.: 2025-07-03). A waiver of informed consent was granted due to the retrospective nature of the analysis.

### Diagnostic and therapeutic strategies

In this study, treatment strategies for pulmonary actinomycosis were individualized based on CT classification and clinical manifestations.

### CT imaging protocol

All scans were performed using a Philips Brilliance 64-slice CT scanner. Protocols included non-contrast chest CT alone or combined non-contrast and contrast-enhanced CT. Multiphanar reconstruction (MPR) images (axial, coronal, sagittal) were independently analyzed by two attending radiologists with associate chief physician qualifications or higher. Scanning parameters: Standard reconstruction algorithm; gantry rotation speed: 0.75 s/rotation; tube voltage: 120 kV; tube current: 250 mA; pitch: 0. 891; reconstruction matrix: 512 × 512 px; slice thickness: 5 mm; slice interval: 5 mm; reconstruction thickness: 1 mm; reconstruction interval: 1 mm. Window Width (WW):lung window: 1500 HU; mediastinal window:350 HU; angiography: 400 HU. Window Level (WL): lung window: -400 HU; mediastinal window: 50 HU; angiography: 60 HU. Contrast protocol: 100 ml non-ionic iodinated contrast medium (iohexol, 350 mgI/ml) administered via right antecubital vein injection at 3 ml/s using a power injector. Scan delays: 25 s (arterial phase) and 100 s (equilibrium phase).

For patients with localized or highly aggressive lesions (e.g., *Airspace suspension type* or *Nodular infiltrative type*), surgical intervention was prioritized. Surgical options included lobectomy, segmentectomy, or pneumonectomy, followed by prolonged intravenous and oral antibiotic therapy (e.g., penicillins, cephalosporins, or fluoroquinolones) to prevent recurrence. For patients with extensive disease or surgical contraindications (e.g., *Bilateral disseminated type* or *Occult manifestation type*), conservative antimicrobial therapy was preferred, involving high-dose intravenous antibiotics followed by oral maintenance therapy. In cases of *Airway-originated type* involvement, endoscopic removal of foreign bodies or broncholiths was performed, combined with antibiotic therapy for infection control. Patients presenting with hemoptysis underwent urgent hemostasis via bronchial artery embolization or intercostal artery embolization. Surgical extent and postoperative management were tailored according to the CT classification during the treatment course. For instance, in trans-lobar or trans-segmental lesions, reinforced suture closure of the bronchial stump and pleural tenting were implemented to mitigate the risk of bronchopleural fistula.

### Statistical analysis

Data analysis was performed using IBM SPSS Statistics version 25. 0 (IBM Corp., Armonk, NY, USA). Categorical variables—including lesion size categories, anatomical location, imaging features, and imaging-based classification—are presented as frequency (percentage). Continuous variables (e.g., mean lesion diameter) underwent normality assessment via the Shapiro-Wilk test (*P >* 0. 05) and homogeneity of variance evaluation using Levene’s test (*P >* 0. 10). When both assumptions were satisfied, data are expressed as mean ± standard deviation (SD), and one-way analysis of variance (ANOVA) was employed. Cohort distribution according to imaging-based classification was: Type I (n = 6), Type II (n = 51), Type III (n = 2), Type IV (n = 6), Type V (n = 4). Given the limited sample size in Type III (n = 2), Fisher’s exact test was universally implemented for all categorical analyses involving this subgroup to ensure methodological rigor. Intergroup comparisons stratified by imaging-based classification (Types I–V) were conducted. Categorical variables were analyzed using Pearson’s chi-square (*χ²*) test, with Fisher’s exact test substitution when any cell expected frequency was <5. Statistical significance was defined as a two-tailed *P* < 0. 05. The “P-value” column denotes results of intergroup comparisons; the symbol “—” indicates statistical non-applicability (e.g., aggregate summary measures or total categorical distributions).

## Results

### Patient characteristics

A total of 69 patients were enrolled in this study. The cohort comprised 57 males (82. 6%) and 12 females (17. 4%), yielding a male-to-female ratio of 4. 8:1. The mean age was 53. 3 ± 12. 1 years. 3 patients (4.3%) were outpatients, while 66 (95.7%) were inpatients. The disease duration ranged from 3 days to 8 years, with a median duration of 2.2 months. The predominant presenting symptoms included cough (n=53, 76.8%) and hemoptysis (n=51, 73. 9%), followed by fever (n=14, 20.3%). Comorbid conditions were present in 28 patients (40. 6%), including tuberculosis, bronchiectasis, chronic obstructive pulmonary disease (COPD), diabetes mellitus, and lung cancer. A history of dental procedures (such as tooth extraction) was reported in 3 patients (4.3%), and 4 patients (5.8%) had a history of poor oral hygiene.

### Chest CT manifestations and classification

Among the chest CT examinations performed, 42 patients underwent contrast-enhanced scans while 27 received non-contrast scans. The mean diameter of solid lesions was 4. 9 ± 1.9 cm. Lesion distribution was as follows: left upper lobe (LUL) in 11 cases (15.9%); LUL and superior segment of left lower lobe in 1 case (1.4%); left lower lobe (LLL) in 10 cases (14.5%); extensive left lung involvement in 2 cases (2.9%); right upper lobe (RUL) in 21 cases (30.4%); right middle lobe (RML) in 4 cases (5.8%); right lower lobe (RLL) in 12 cases (17.4%); right upper and middle lobes in 3 cases (4.3%); extensive right lung involvement in 3 cases (4.3%); and bilateral extensive involvement in 2 cases (2.9%).

**Figure 1:**
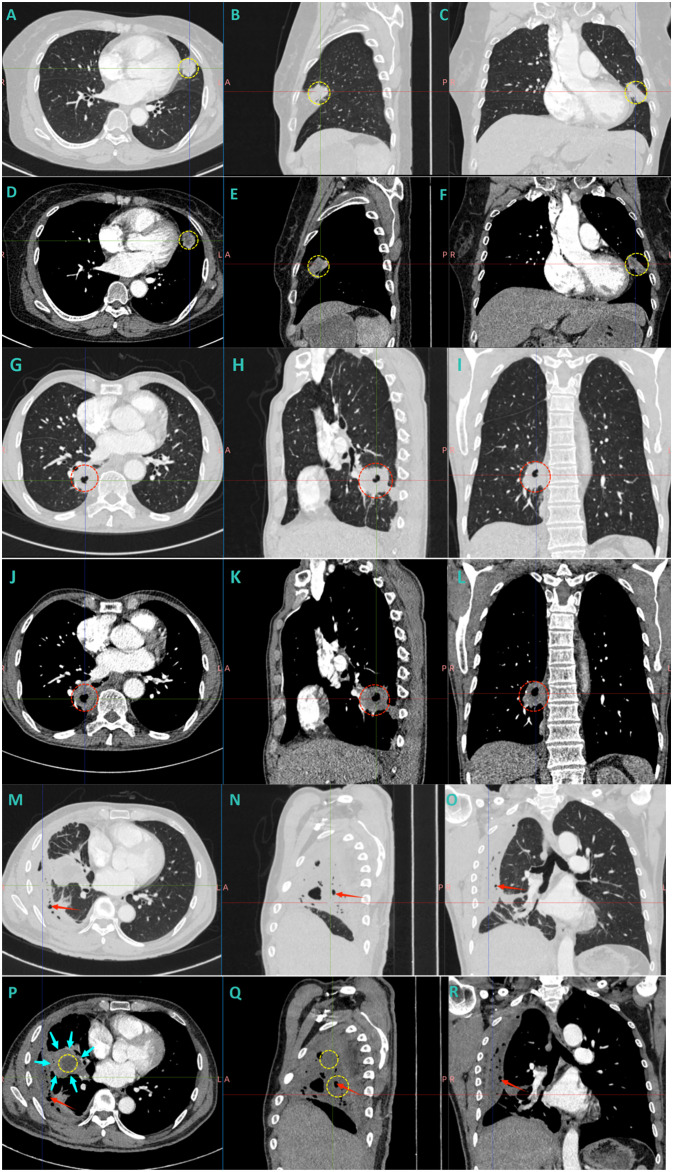
Necrosis, liquefaction, consolidation, airspace suspension sign and cavitation sign. Case 1. Images **(A-C)** (lung window, WW: 1500 HU; WL: -400 HU)| **(D-F)** (mediastinal window, WW: 350 HU; WL: 50 HU): Clinical History: A 63-year-old woman presented with cough for 9 months and hemoptysis for more than 3 months. Chest CT Findings:A mass-like consolidation measuring 17. 00 mm × 26. 00 mm was observed in the lingular segment of the left upper lobe [yellow circles in **(A–F)**]. Contrast-enhanced imaging revealed heterogeneous moderate enhancement with internal calcified nodules. Adjacent pleural adhesion was noted. Initial Misdiagnosis: Lung carcinoma. Duration of Misdiagnosis: More than 4 months. Definitive Diagnosis: CT-guided core needle biopsy. Treatment: Non-surgical management. Therapeutic Agents: Levofloxacin, Rifampin, Amoxicillin. Treatment Duration: 20 months. Therapeutic Outcome: Complete resolution. Case 2. Images **(G-I)** [lung window, WW: 1500 HU; WL: -400 HU)| **(J-L)** (mediastinal window, WW: 350 HU; WL: 50 HU]: Clinical History:A 69-year-old man presented with cough, expectoration, and hemoptysis for 3 months. Chest CT Findings: An irregular mass measuring 34. 90 mm × 26. 60 mm with nodular components was identified in the right lower lobe, demonstrating internal cavitation (*Cavitation Sign)* [red circle in **(G–L)**]. Contrast-enhanced imaging revealed marked heterogeneous enhancement. Necrotic foci were present within the lesion, accompanied by a small pleural effusion, pleural thickening, and adhesions. Enlargement of the right hilar lymph nodes was noted. Initial Misdiagnosis: Lung abscess. Duration of Misdiagnosis: 3 months. Definitive Diagnosis: Histopathology of surgical specimen. Treatment: Surgical intervention was performed, followed by postoperative antibiotic therapy. After discharge, penicillin-based maintenance antibiotic therapy was continued for 9 months. Surgical procedure: Video-assisted thoracoscopic surgery (VATS) right lower lobectomy. Postoperative Complications:Empyema; Atelectasis of residual lung. Complication Management: Continuation of antibiotic therapy and implementation of pulmonary rehabilitation maneuvers. Treatment Duration: 10 months. Therapeutic Outcome: Cured. Case 3. Images **(M-O)** (lung window, WW: 1500 HU; WL: -400 HU)| **(P-R)** (mediastinal window, WW: 350 HU; WL: 50 HU): Clinical History: A 51-year-old man presented with cough, expectoration, and chest tightness for more than 3 months. Chest CT Findings: An irregular mass measuring 59 × 48 mm was identified within the lateral segment of the right middle lobe, exhibiting heterogeneous hypodensity on unenhanced imaging. Post-contrast scans demonstrated marked heterogeneous enhancement, featuring a central non-enhancing hypodense area indicative of liquefied necrosis [yellow circles in **(P, Q)**], surrounded by peripheral irregular enhancement and consolidation [blue arrows in **(P)**]. Obstruction of the lateral segmental bronchus was identified. Diffuse right pleural thickening and crescent-shaped loculated pleural effusion containing irregular air collections (*Airspace suspension Sign*) (red arrows in **(M–R)** were observed. The pleura exhibited intense enhancement on contrast-enhanced scans. Initial Misdiagnosis: Organizing pneumonia. Duration of Misdiagnosis: 3 months. Definitive Diagnosis: Histopathology of surgical specimen. Treatment: Treatment regimen: Surgical intervention was performed, followed by postoperative antimicrobial therapy. After discharge, maintenance therapy with amoxicillin was continued for 10 months. Surgical procedure: Right middle lobectomy + right lower lobe wedge resection + decortication of pleural fibrotic plaque. Postoperative Complications: None. Treatment Duration: more than1 year. Therapeutic Outcome: Cured.

**Figure 2:**
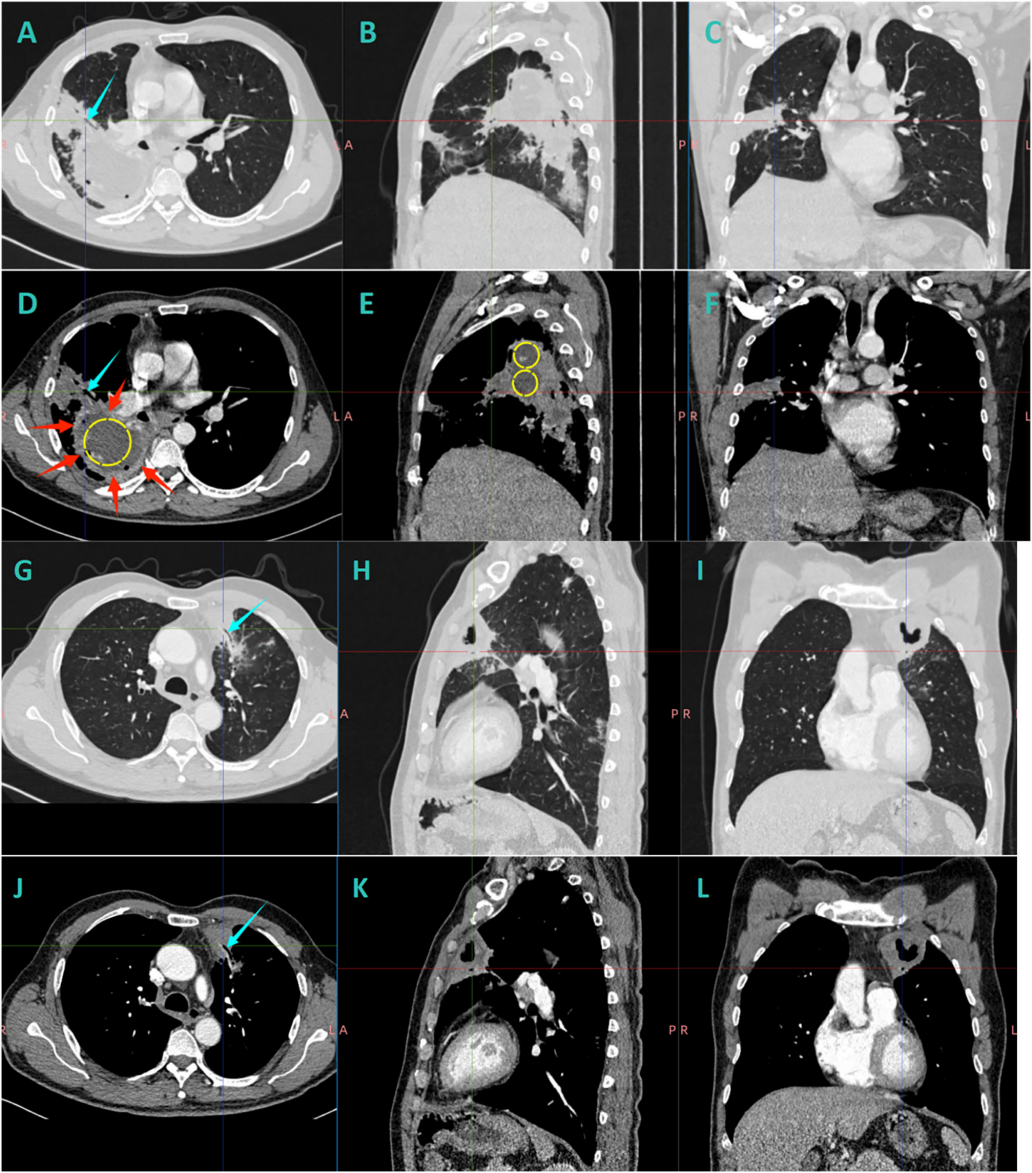
Necrosis, liquefaction, consolidation, air bronchogram sign, and bronchial cutoff sign. Case 1. Images **(A-C)** (lung window, WW: 1500 HU; WL: -400 HU)| **(D-F)** (mediastinal window, WW: 350 HU; WL: 50 HU): Clinical History: A 47-year-old man presented with intermittent cough for 1 year, exacerbated over 3 months. Chest CT Findings: A giant mass measuring 81. 80 mm × 71. 30 mm with vessel penetration was identified in the right upper lobe and dorsal segment of the right lower lobe. The lesion demonstrated liquefactive necrotic hypodense areas (yellow circles in **(D, E)** (CT attenuation: -54. 00 to 73. 00 HU, mean 2. 96 HU) and multiple air-containing cavities. Absence of significant enhancement was noted centrally, while the peripheral consolidated component exhibits rim enhancement [red arrows in **(D)**] (CT attenuation: 20. 00 to 174. 00 HU, mean 89. 33 HU). Focal atelectasis with *Air bronchograms sign* [blue arrows in **(A, D)**] was observed in the right upper lobe, accompanied by right pleural thickening. Initial Misdiagnosis: Pulmonary tuberculosis. Duration of Misdiagnosis: more than 1 year. Definitive Diagnosis: Pathological biopsy of bronchoscopically obtained specimens Treatment: Non-surgical management. Therapeutic Agents:Moxifloxacin, Penicillin G, Amikacin, Tinidazole. Treatment Duration: 16 months. Therapeutic Outcome: Complete resolution. Case 2. Images **(G-I)** (lung window, WW: 1500 HU; WL: -400 HU)| **(J-L)** (mediastinal window, WW: 350 HU; WL: 50 HU): Clinical History: A 59-year-old man presented with fever, cough, and hemoptysis for over 3 weeks. Chest CT Findings: An irregular soft-tissue mass subjacent to the anterior chest wall was observed in the anterior segment of the left upper lobe. The lesion demonstrated focal lucencies and air bronchograms, with *Bronchial Cutoff Sign* of the distal anterior segmental bronchus [blue arrows in **(G, J)**]. Initial Misdiagnosis: Lung carcinoma. Duration of Misdiagnosis: 3 weeks. Definitive Diagnosis: Histopathology of surgical specimen. Treatment: Surgical intervention was performed, followed by postoperative antibiotic therapy. After discharge, penicillin-based maintenance antibiotic therapy was continued for 10 months. Surgical procedure: Left upper lobectomy. Postoperative Complications:Empyema; Atelectasis of residual lung. Management of Complications: Hospital readmission was initiated, with subsequent performance of thoracic tube drainage and intensified antibiotic therapy, while conducting pulmonary rehabilitation maneuvers. Treatment Duration: More than 12 months. Therapeutic Outcome: Complete resolution.

**Figure 3:**
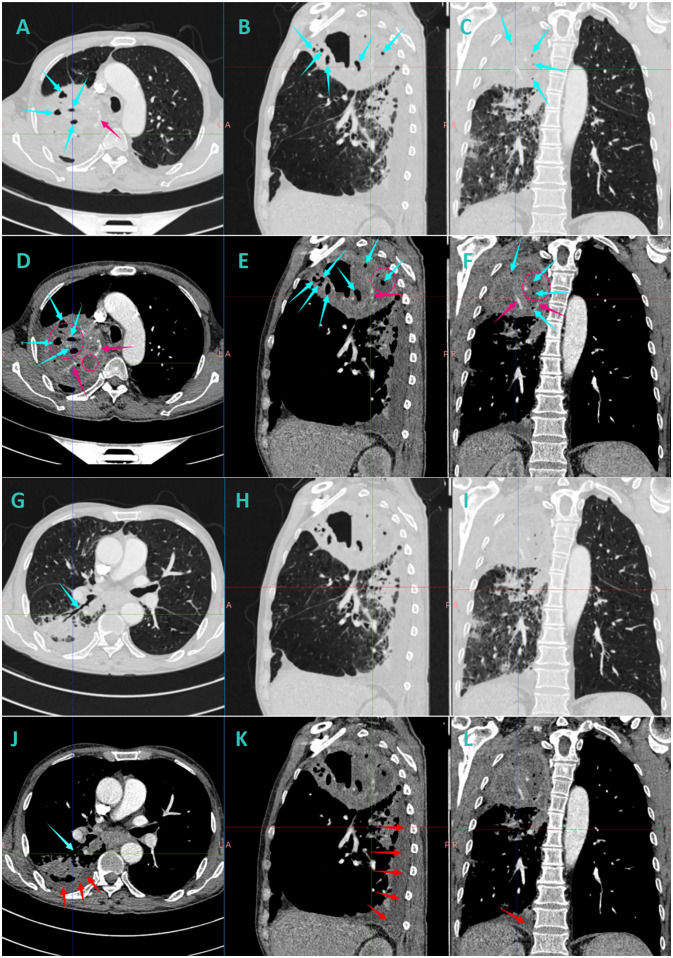
Classification of chest CT findings: type II: airspace suspension type. Images **(A-C, G-I)** (lung window, WW: 1500 HU; WL: -400 HU)| **(D-F, J-L)** (mediastinal window, WW: 350 HU; WL: 50 HU): Clinical History: A 65-year-old man presented with hemoptysis accompanied by cough and expectoration for over 5 months. Chest CT Findings: There was occlusion of the right upper lobe segmental bronchus and enlargement of the right hilum. A large mass measuring 90. 00 mm × 88. 57 mm was observed in the right upper lobe. Within the mass, areas of liquefactive necrosis [red circles in **(D–F)**] and multiple air-containing cavities (*Airspace suspension Sign*) [blue arrows in **(A–F)**] were identified (CT attenuation values: -69. 00 HU to 88. 00 HU, mean 4. 74 HU). On contrast-enhanced scan, the periphery of the mass demonstrated septal enhancement, while the liquefactive necrosis areas showed no enhancement (CT attenuation values: 13. 00 HU to 90. 00 HU, mean 57. 16 HU). Right upper pulmonary vessels were seen traversing the mass [red arrows in **(A, D–F)**] without evidence of obvious invasion. Multiple high-attenuation patchy opacities, containing air bronchograms (*Air bronchogram sign*) [blue arrows in **(G, J)**], were present in the right lower lobe, with peripheral reticular changes. A crescent-shaped fluid-attenuation shadow [red arrows in **(J–L)**] was noted in the right pleural cavity. Enlarged lymph nodes were identified in stations 2R, 4, and 7. Initial Misdiagnosis: Pulmonary tuberculosis. Duration of Misdiagnosis: More than 5 months. Definitive Diagnosis: Pathological biopsy of bronchoscopically obtained specimens. Treatment: Non-surgical management. Therapeutic Agents: Cephalosporins, Penicillin G, Amoxicillin. Treatment Duration: More than 12 months. Therapeutic Outcome: Complete resolution.

**Figure 4:**
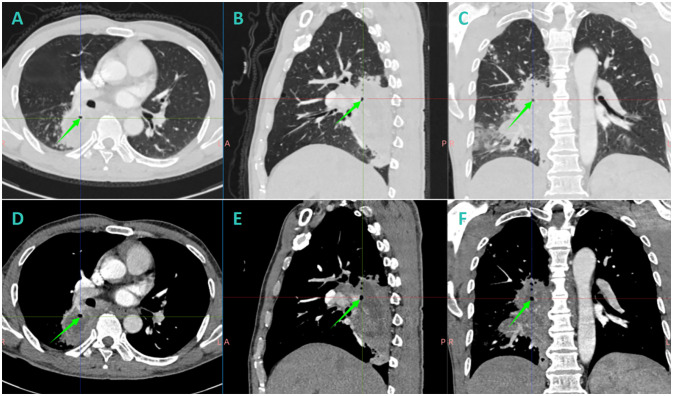
Classification of chest CT findings: type II: airspace suspension type. Images **(A-C)** (lung window, WW: 1500 HU; WL: -400 HU)| **(D-F)** (mediastinal window, WW: 350 HU; WL: 50 HU): Clinical History: A 49-year-old man presented with intermittent cough and hemoptysis for over 2 months. Chest CT Findings: Left lung (posterior segment of upper lobe; dorsal and basal segments of lower lobe): Ill-defined masses were present in the posterior segment of the left upper lobe and the apical and basal segments of the left lower lobe, with local extension across the fissure. Occlusion was noted in the apical segment of the right lower lobe. The lesions exhibited heterogeneous attenuation with patchy low-attenuation areas (CT attenuation values: -48. 00 HU to 52. 00 HU, mean 8. 77 HU). Adjacent pleural thickening with adhesion was observed. Contrast-enhanced scanning revealed fine vascular structures traversing the lesions, with heterogeneous mild peripheral enhancement (CT attenuation values: 22. 00 HU to 130. 00 HU, mean 70. 10 HU). Non-enhancing central areas, including cavitation [*Airspace suspension Sign*) (green arrows in **(A–F)**], were identified within the lesions. Scattered enlarged mediastinal lymph nodes were noted, demonstrating homogeneous and marked enhancement post-contrast. Initial Misdiagnosis: Lung carcinoma. Duration of Misdiagnosis: More than 2 months. Definitive Diagnosis: Pathological biopsy of bronchoscopically obtained specimens. Treatment: During hospitalization, the patient underwent “Bronchial Artery Embolization (BAE)” for massive hemoptysis and received anti-infective therapy. Following discharge, oral amoxicillin therapy was continued for anti-infective purposes for over 1. 5 years. Therapeutic Agents: Cefoperazone-Sulbactam, Penicillin G, Amoxicillin. Treatment Duration: More than 1. 5 years. Therapeutic Outcome: Complete resolution.

**Figure 5:**
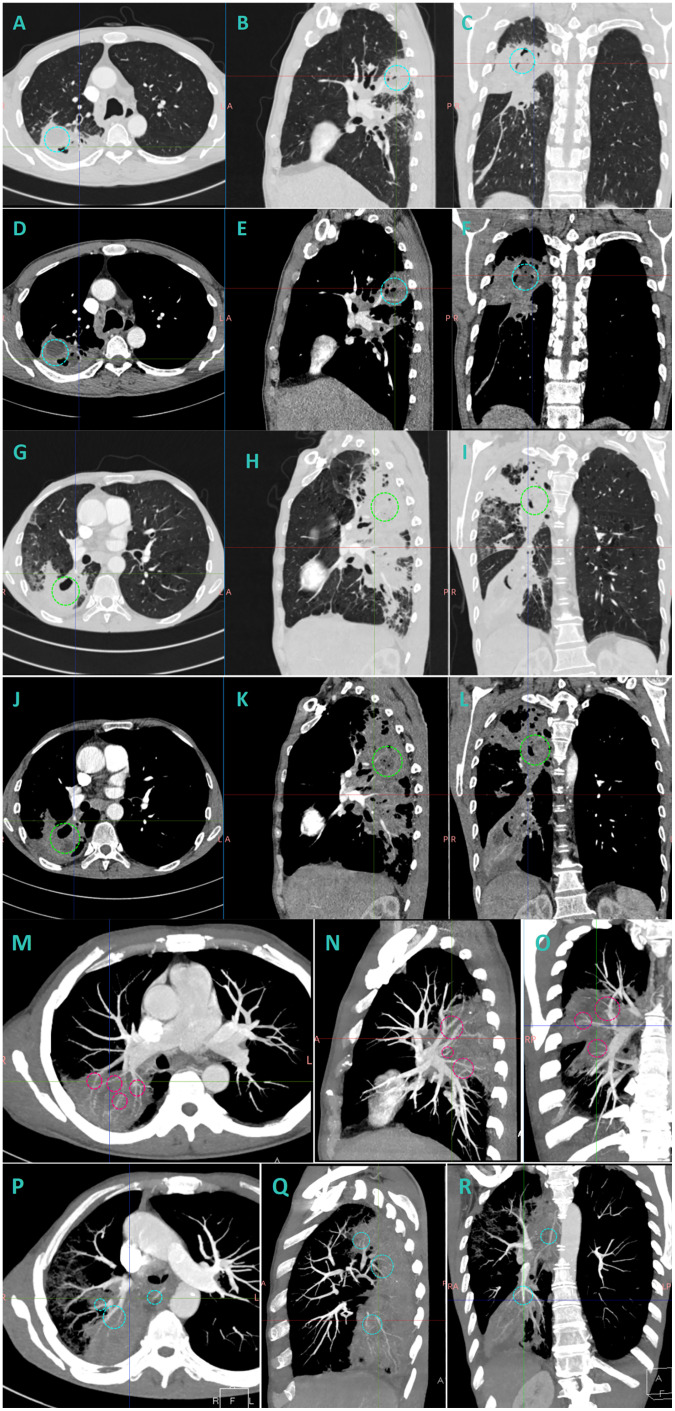
Central necrosis with liquefactive change, vascular traversing sign. Case 1. Images **(A-C)** (lung window, WW: 1500 HU; WL: -400 HU) **(D-F)** (mediastinal window, WW: 350 HU; WL: 50 HU)| **(M-O)** (angiography, WW: 400 HU; WL: 60 HU): Clinical History: A 47-year-old man presented with cough, expectoration, and fever for 6 months. Chest CT Findings: An irregular mass measuring approximately 62. 00 mm × 48. 00 mm was observed in the posterior segment of the right upper lobe, extending to the dorsal segment of the right lower lobe. The mass exhibited soft-tissue attenuation with areas of liquefactive necrosis (*Central necrosis with liquefactive change*) (blue circles in **(A-F)**. On contrast-enhanced imaging, the mass showed heterogeneous enhancement, while the necrotic areas remained non-enhancing. The mass encased the right upper and lower pulmonary arteries (*Vascular traversing sign*) (red circles in **(M-O)**). Initial Misdiagnosis: Pneumonia. Duration of Misdiagnosis: 6 months. Definitive Diagnosis: Pathological biopsy of bronchoscopically obtained specimens. Treatment: Non-surgical management. Therapeutic Agents:Penicillin G, Piperacillin-tazobactam, Amoxicillin. Treatment Duration: 18 months. Therapeutic Outcome: Significant improvement. Case 2. Images **(G-I)** (lung window, WW: 1500 HU; WL: -400 HU)|**(J-L)** (mediastinal window, WW: 350 HU; WL: 50 HU)| **(P-R)** (angiography, WW: 400 HU; WL: 60 HU): Clinical History: A 63-year-old man presented with intermittent cough and expectoration for more than1 year. Chest CT Findings: An irregular mass measuring 62. 00 mm × 48. 00 mm was noted in the posterior segment of the right upper lobe, extending to the dorsal segment of the right lower lobe. The lesion demonstrated soft-tissue attenuation with internal areas of liquefactive necrosisblack (*Central necrosis with liquefactive change*) (green circles in **(G-L)**). On contrast-enhanced images, the mass exhibited heterogeneous enhancement, while the necrotic areas showed no enhancement. The mass encased the right upper and lower pulmonary arteries (*Intralesional Traversing Vessel Sign)* (blue circles in **(P-R)**). Initial Misdiagnosis: Pneumonia. Duration of Misdiagnosis: 1 year. Definitive Diagnosis: Pathological biopsy of bronchoscopically obtained specimens. Treatment: Non-surgical management. Therapeutic Agent: Penicillin G, Amoxicillin. Treatment Duration: 18 months. Therapeutic Outcome: Significant improvement.

CT manifestations included: central necrosis with liquefaction, perilesional consolidation, pulmonary nodules (**Figures 1A–F**), pulmonary cavity (**Figures 1G–L**), thickening of interlobular septa or fissures, atelectasis, air bronchograms (**Figures 2A–F**), bronchial stenosis, occlusion, or Bronchial cutoff sign (**Figures 2G–L**), Intralesional airspace/air-containing cavity/Airspace suspension Sign (**Figures 1M–R**, **3A–F**, **4A–F**), pleural thickening, mediastinal lymph node enlargement, pericardial effusion, pleural effusion, empyema (**Figures 1M–R**), and vascular traversing sign (**Figures 5M–R**).

On contrast-enhanced CT scans (arterial and equilibrium phases), the perilesional consolidation exhibited ring-like or heterogeneous enhancement in the majority of cases, with only 3 cases demonstrating homogeneous enhancement. The central hypodense necrotic/liquefied areas showed Hounsfield Unit (HU) values ranging from -87. 00 to 88. 00 HU; enhancement was observed in only 1 case, with absence of enhancement in all others. The surrounding soft tissue attenuation areas and cavity walls exhibited HU values between 2. 00 and 225. 00 HU, demonstrating marked enhancement with an average increase of approximately 50. 00 HU (range: 15. 00-73. 00 HU). Traversing, displacement, encasement, or traction of vessels within lesions was observed in 30 patients (**Figures 5M–R**).

Based on these characteristics (**Table 1**) and relevant literature, the CT findings were classified into the following types: Type I: Nodular infiltrative type (8. 7%) (**Figure 6**); Type II: Airspace suspension type (73. 9%) (**Figures 3**-**7**); Type III: Bilateral disseminated type (2. 9%) (**Figure 8**); Type IV: Airway-originated type (8. 7%) (**Figures 9**-**11**); Type V: Occult manifestation type (5. 8%) (**Figures 12**-**14**).

**Table 1:** Computed tomography (CT) features and CT-based classification of pulmonary actinomycosis *(N=69)*.

Feature	Value
Lesion size (cm)	
< 3	8 (11. 6)
3 - 4. 9	17 (24. 6)
5 - 6. 9	20 (29. 0)
7 - 8. 9	11 (15. 9)
≥ 9	4 (5. 8)
Non-measurable lesions*	9 (13. 0)
Mean lesion diameter, cm (SD)	4. 9 ± 1. 9
Location	
Left upper lobe	11 (15. 9)
Left upper lobe + lower lobe (dorsal segment)	1 (1. 4)
Left lower lobe	11 (15. 9)
Diffuse left lung	2 (2. 9)
Right upper lobe	20 (29. 0)
Right middle lobe	4 (5. 8)
Right upper + middle lobes	3 (4. 3)
Right lower lobe	12 (17. 4)
Diffuse right lung	3 (4. 3)
Bilateral diffuse lesions	2 (2. 9)
Imaging sign	
Pulmonary nodules/Interlobular septal or fissure thickening	22 (31. 9)
Central necrosis with liquefactive change	55 (79. 7)
Pulmonary cavity	28 (40. 6)
Perilesional consolidation	45 (65. 2)
Perilesional rim or heterogeneous enhancement	33 (47. 8)
Perilesional homogeneous enhancement	5 (7. 2)
Atelectasis/Air bronchogram sign	53 (76. 8)
Intralesional airspace/Air-containing cavity/Airspace suspension Sign	45 (65. 2)
Radiologic pleural thickening	36 (52. 2)
Mediastinal lymph node enlargement	47 (68. 1)
Loculated pleural effusion/Free-flowing pleural effusion	12 (17. 4)
Bronchiectasis	11 (15. 9)
Bronchial stenosis/Bronchial obstruction/Bronchial cutoff sign	19 (27. 5)
Spiculation	20 (29. 0)
Lobulation	16 (23. 2)
Pleural retraction	16 (23. 2)
Intralesional traversing vessel sign/Vascular traversing sign	30 (43. 5)
Chest wall invasion/Sinus tract formation	1 (1. 4)
Radiologic pericardial thickening/Pericardial effusion	3 (4. 3)
Imaging classification	
Type I: Nodular infiltrative type	6 (8. 7)
Type II: Airspace suspension type	51 (73. 9)
Type III: Bilateral disseminated type	2 (2. 9)
Type IV: Airway-originated type	6 (8. 7)
Type V: Occult manifestation type	4 (5. 8)

Includes non-solid, scattered, or diffusely distributed lesions (marked with*).

**Figure 6:**
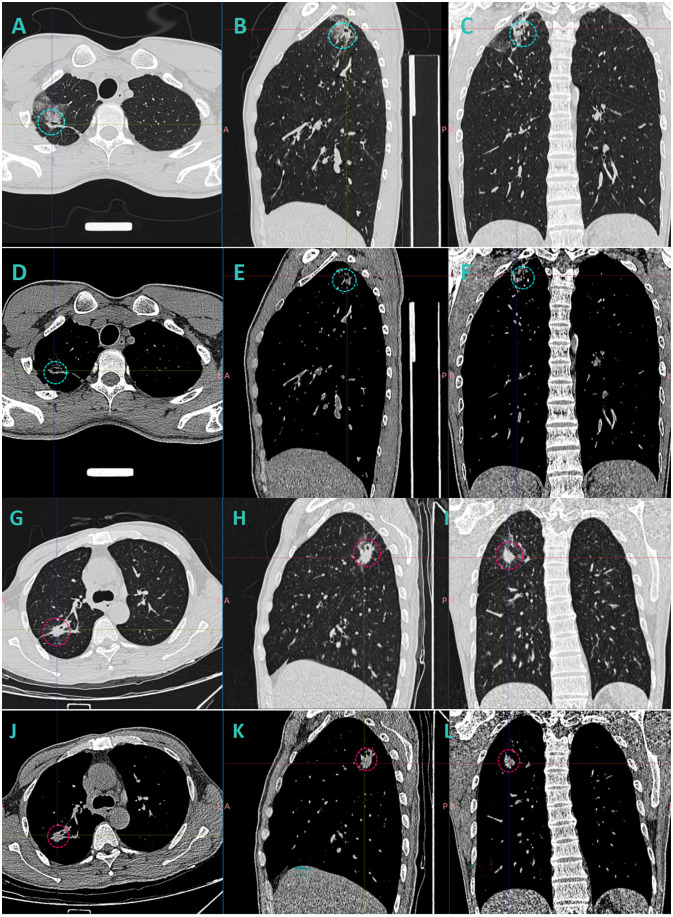
Classification of chest CT findings: type I: nodular-infiltrative type. Case 1. Images **(A-C)** (lung window, WW: 1500 HU; WL: -400 HU)| **(D-F)** (mediastinal window, WW: 350 HU; WL: 50 HU): Clinical History: A 27-year-old man presented with intermittent cough and hemoptysis for over 2 years. Chest CT Findings: Irregular patchy opacities and nodular lesions with speckled calcifications were observed in the apical and posterior segments of the right upper lobe. Peripheral features compatible with aspiration pneumonia were noted, while focal hypodense areas and small cavity (blue circles in **(A–F)**) were identified within the lesions. Initial Misdiagnosis: Pulmonary tuberculosis. Duration of Misdiagnosis: More than 2 years. Definitive Diagnosis: Histopathology of surgical specimen. Treatment: Surgical intervention was performed, followed by postoperative intravenous cephalosporins for 2 weeks. No maintenance antibiotic therapy after discharge. Surgical procedure: Video-assisted thoracoscopic surgery (VATS) right upper lobectomy. Postoperative Complications: Atelectasis of the residual lung, thoracic infection. Management of Complications: Thoracic drainage, antibiotic therapy and respiratory rehabilitation. Treatment Duration: More than1 month. Therapeutic Outcome: Complete resolution. Case 2. Images **(G-I)** (lung window, WW: 1500 HU; WL: -400 HU)| **(J-L)** (mediastinal window, WW: 350 HU; WL: 50 HU): Clinical History: A 51-year-old man presented with intermittent hemoptysis exceeding 5 years. Chest CT Findings: An irregular soft-tissue attenuation lesion measuring 20. 22 mm × 20. 00 mm was identified in the posterior segment of the right upper lobe. The mass demonstrated internal air-containing lacunae and exhibited lobulated contours, spiculated margins, and pleural retraction, with associated thickening of the interlobar fissure. Focal hypodense liquefactive necrosis with small cavities was noted within the lesion (red circles in **(G–L)**). Initial Misdiagnosis: Pulmonary tuberculosis. Duration of Misdiagnosis: More than 5 years. Definitive Diagnosis: Histopathology of surgical specimen. Treatment: Surgical intervention was performed, followed by postoperative. intravenous cephalosporins for 2 weeks. Maintenance oral antibiotics for 13 months post-discharge. Surgical procedure: Right upper lobectomy. Postoperative Complications: None. Treatment Duration: More than 1 year. Therapeutic Outcome: Complete resolution.

**Figure 7:**
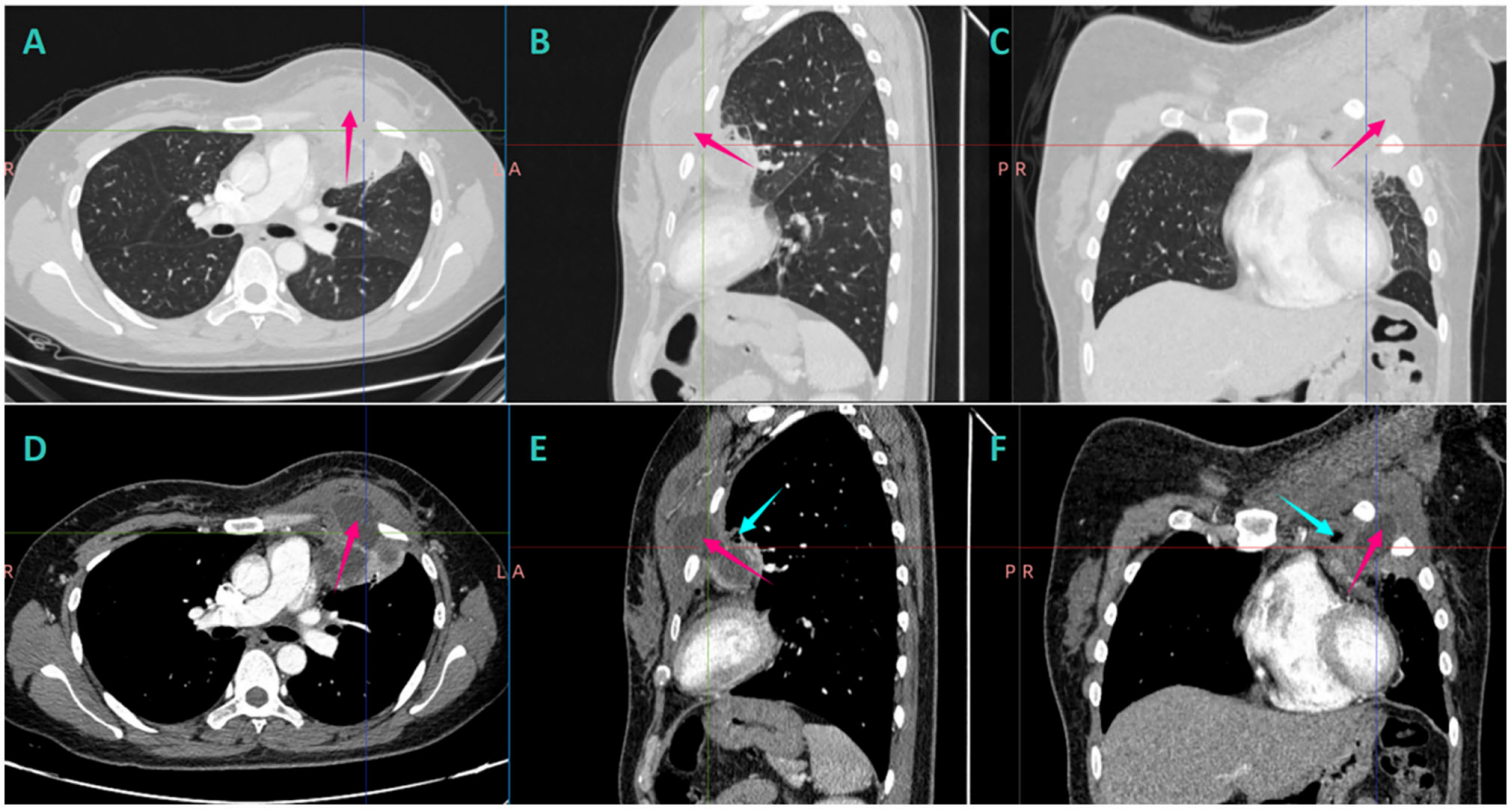
Classification of chest CT findings: type II: airspace suspension type. Images **(A-C)** (lung window, WW: 1500 HU; Window level: -400 HU)| **(D-F)** (mediastinal window, WW: 350 HU; WL: 50 HU): Clinical History: A 33-year-old woman presented with chest pain for 2 years and intermittent hemoptysis for 6 months. Chest CT Findings: An irregular mass-like consolidation was present in the anterior segment and lingula of the left upper lobe, demonstrating heterogeneous attenuation. Post-contrast imaging revealed heterogeneous rim enhancement (CT attenuation values: 47. 00 HU to 103. 00 HU, mean 75. 00 HU). A central low-attenuation area was identified within the lesion (CT attenuation values: 53. 00 HU to 144. 00 HU, mean 106. 56 HU), containing a small air cavity (*Airspace suspension Sign*) (blue arrows in **(E, F)**). Soft tissue swelling with multiple low-attenuation areas was noted surrounding the left anterior second and third ribs, demonstrating rim enhancement post-contrast. The mass in the left upper lobe was contiguous with the chest wall mass (red arrows in **(A–F)**). A minimal amount of fluid-attenuation material was present within the pericardial space. Initial Misdiagnosis: Pulmonary tuberculosis and chest wall tuberculosis. Duration of Misdiagnosis: Approximately 2 years. Definitive Diagnosis: Histopathology of surgical specimen. Treatment: Surgical intervention was performed, followed by postoperative penicillin therapy for 2 weeks to prevent infection. Upon discharge, maintenance antimicrobial therapy with amoxicillin was continued for 11 months. Surgical Procedure: Left upper lobectomy with en bloc debridement of chest wall abscess. Postoperative Complications: None. Total Treatment Duration: 12 months. Therapeutic Outcome: Complete resolution.

**Figure 8:**
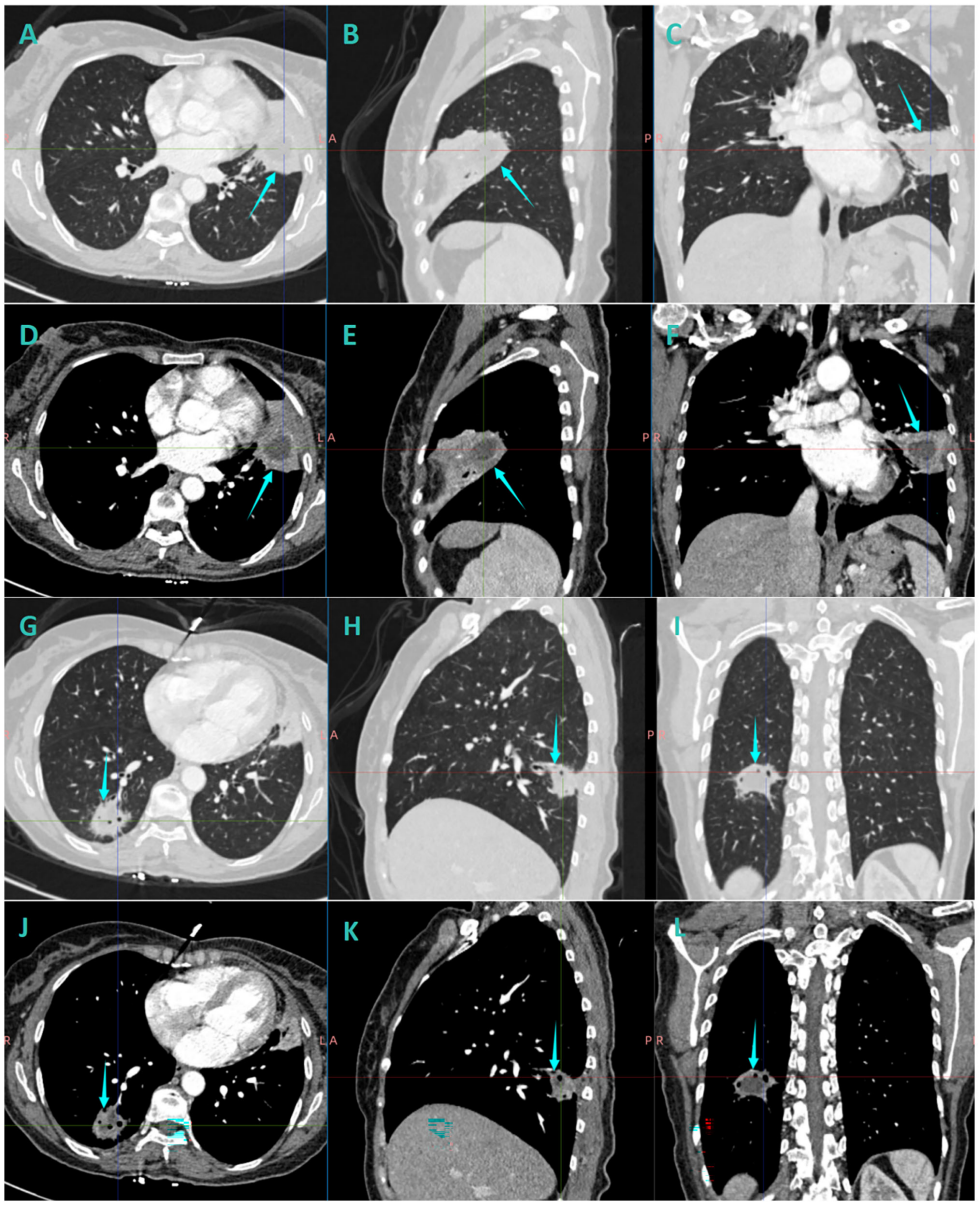
Classification of chest CT findings: type III: bilateral disseminated type. Images **(A-C, G-I)** (lung window, WW: 1500 HU; WL: -400 HU)| **(D-F, J-L)** (mediastinal window, WW: 350 HU; WL: 50 HU): Clinical History: A 51-year-old woman presented with intermittent cough for 4 months and hemoptysis persisting for over 2 months. Chest CT Findings: An oval-shaped solid mass, measuring approximately 33. 30 mm × 24. 40 mm, was seen in the subpleural region of the posterior basal segment of the right lower lobe (blue arrows in **(G–L)**). The mass demonstrated a small cavity internally, spiculated margins, and surrounding hazy linear opacities. The draining bronchus was obstructed. On contrast-enhanced scan, the lesion exhibited rim enhancement (CT attenuation values: 47. 00 HU to 103. 00 HU, mean 75. 00 HU) with a central liquefactive necrosis area (CT attenuation values: -45. 00 HU to 41. 00 HU, mean -1. 28 HU). In the lingular segment of the left upper lobe, the bronchus was severely narrowed to near occlusion. A solid mass-like consolidation, measuring approximately 57. 30 mm × 58. 25 mm with indistinct margins, was present. Post-contrast imaging showed homogeneous enhancement of the lesion (CT attenuation values: 42. 00 HU to 120. 00 HU, mean 85. 23 HU) containing a central low-attenuation necrotic area (CT attenuation values: -38. 00 HU to 57. 00 HU, mean 13. 42 HU) (blue arrows in **(A–F)**. A small amount of pleural fluid was noted in the right hemithorax. Initial Misdiagnosis: Lung carcinoma. Duration of Misdiagnosis: More than 2 months. Definitive Diagnosis: CT-guided percutaneous lung biopsy with specimen acquisition for pathological examination. Treatment: The patient was hospitalized twice. During the first admission, Bronchial Artery Embolization (BAE) was performed for massive hemoptysis. During the second admission, embolization of multiple systemic collateral arteries was performed, also for massive hemoptysis. Anti-infective therapy was administered during both hospitalizations. Following discharge, oral amoxicillin therapy was continued for over 1 year. Therapeutic Agents: Levofloxacin, Penicillin G, Amoxicillin. Total Treatment Duration: More than 1. 5 years. Therapeutic Outcome: Significant clinical and radiographic improvement.

**Figure 9:**
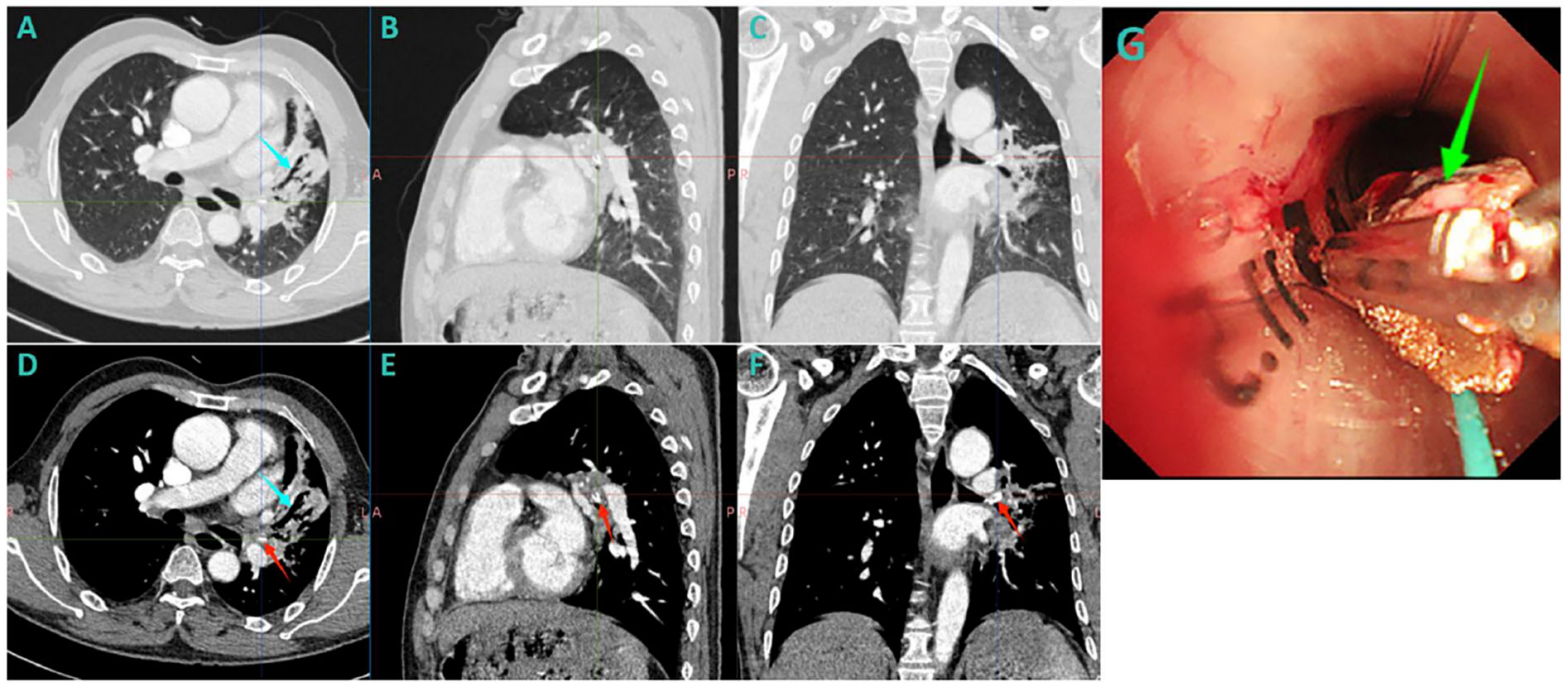
Classification of chest CT findings: type IV: airway-originated type. Clinical History: A 54-year-old man presented with intermittent chest tightness and palpitations for 2 years, worsening over the preceding 3 months. Chest CT and Bronchoscopic Findings: Images **(A-C)** (lung window, WW: 1500 HU; WL: -400 HU)| **(D-F)** (mediastinal window, WW: 350 HU; WL: 50 HU): A hyperdense focus, suggestive of a foreign body, was seen within the left upper lobar bronchus (red arrows in **(D–F)**). Multiple nodules, patchy opacities, and consolidations were present in the left lung. Bronchi were visualized traversing these lesions (blue arrows in **(A, D)**). Post-contrast imaging demonstrated marked homogeneous enhancement of these areas. An irregular hyperdense shadow was identified within the left upper lobe bronchus (red arrows in **(D–F)**). Image G (Bronchoscopy): An irregular foreign body was found impacted in the lumen of the left upper lobe bronchus. A ”chicken bone-like” foreign body (green arrow in **(G)**) was extracted under direct visualization. Initial Misdiagnosis: Pneumonia. Duration of Misdiagnosis: 2 years. Definitive Diagnosis: Pathological biopsy of bronchoscopically obtained specimens Treatment: The ”chicken bone-like” foreign body was extracted via bronchoscopy under direct visualization, followed by anti-infective therapy during hospitalization. Oral amoxicillin therapy was continued for over one year following discharge. Therapeutic Agents: Cephalosporins, Levofloxacin, Penicillin G, Amoxicillin. Treatment Duration: More than 1 year. Therapeutic Outcome: Complete resolution.

**Figure 10:**
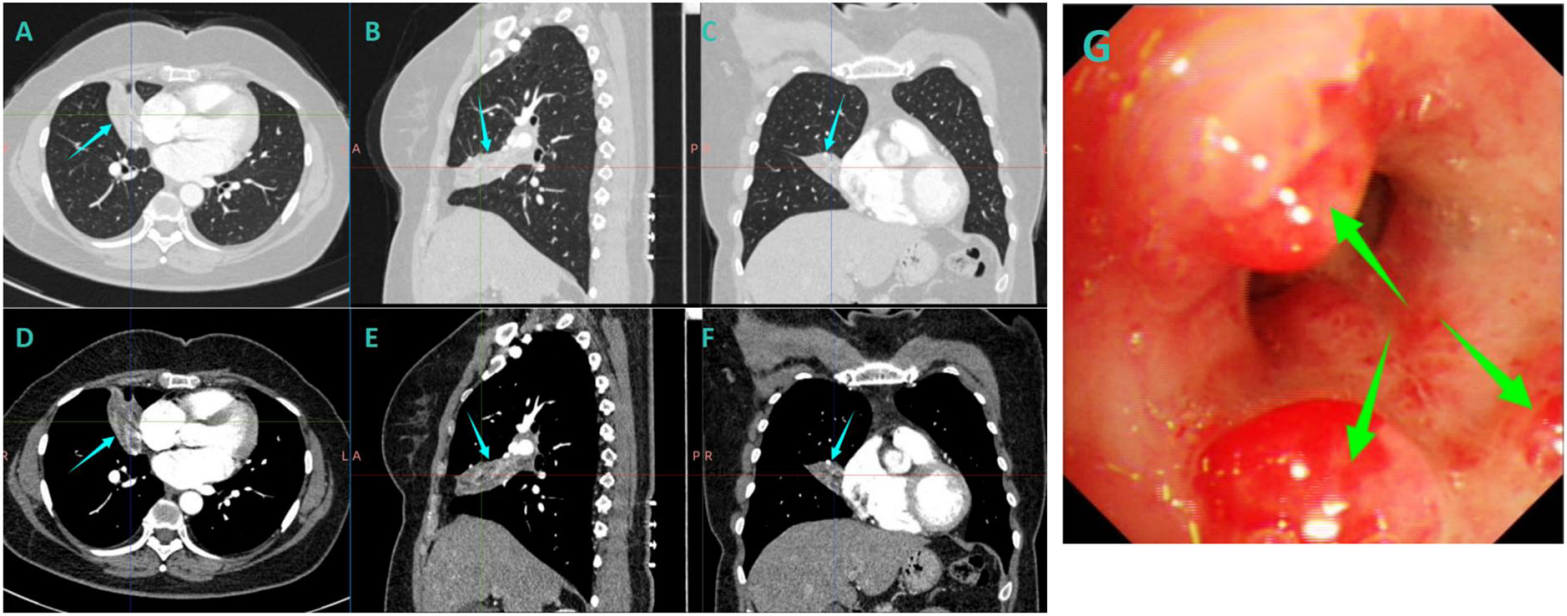
Classification of chest CT findings: type IV: airway-originated type. Clinical History: A 44-year-old woman presented with recurrent cough and expectoration for 5 months. Chest CT and Bronchoscopic Findings: Images **(A-C)** (lung window, WW: 1500 HU; WL: -400 HU)| **(D-F)** (mediastinal window, WW: 350 HU; WL: 50 HU): The right hilum was slightly enlarged. Occlusion of the middle lobe bronchus was observed, with volume loss in the right middle lobe presenting as a mass-like consolidation with irregular margins (blue arrows in **(A–F)**). Image **(G)** (Bronchoscopy): The orifice of the right middle lobe bronchus appeared narrowed, forming a slit-like opening, with erythematous and mildly thickened mucosa. Three nodular proliferations of varying sizes were noted in the lumen of the right middle lobe (green arrows in **(G)**). Initial Misdiagnosis: Pulmonary tuberculosis. Duration of Misdiagnosis: 5 months. Definitive Diagnosis: Pathological biopsy of bronchoscopically obtained specimens Treatment: The lesion was resected via bronchoscopy under direct visualization, accompanied by anti-infective therapy during hospitalization. Oral amoxicillin therapy was continued for over 1 year following discharge. Therapeutic Agent: Penicillin G, Amoxicillin. Treatment Duration: More than 1 year. Therapeutic Outcome: Cured.

**Figure 11:**
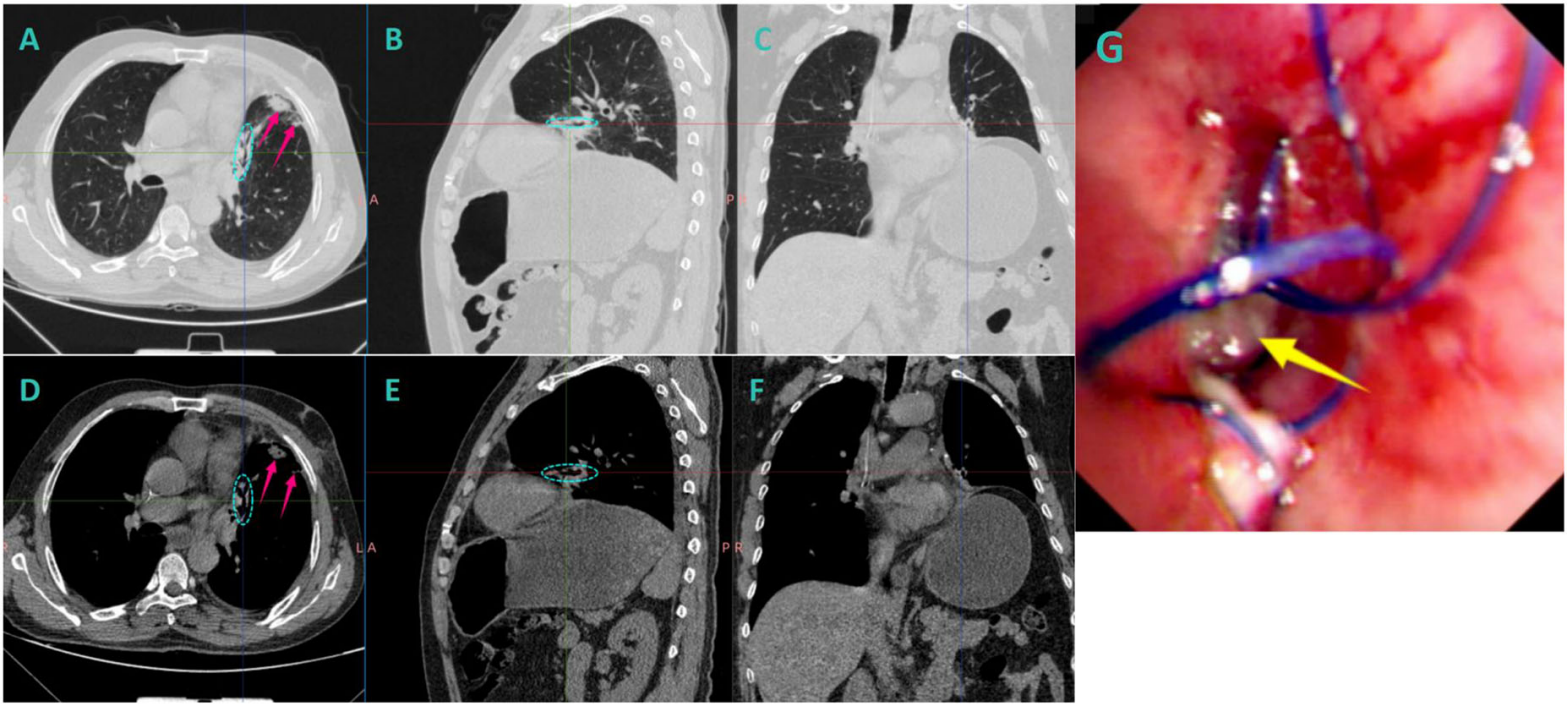
Classification of chest CT findings: type IV: airway-originated type. Clinical History: A 54-year-old man presented with cough for over 1 week, 1 month after left upper lobectomy for squamous cell carcinoma. CT and Bronchoscopic Findings: Images **(A-C)** (lung window, WW: 1500 HU; WL: -400 HU)| **(D-F)** (mediastinal window, WW: 350 HU; WL: 50 HU): Left lung infection with focal atelectasis was observed blue ovals in **(A, B, D, E)**. Multiple punctate, patchy, and linear opacities were visible [red arrows in **(A, D)**]. Image **(G)** (Bronchoscopy): The mucosa of the left upper lobe bronchial stump appeared erythematous, edematous, and hypertrophic. Stenosis was noted at the orifice of the left lower lobe bronchus. A clump-like white purulent material [yellow arrow in **(G)**] was seen, connected to a blue suture at its inferior aspect. Initial Misdiagnosis: Pneumonia and bronchial stump inflammation. Duration of Misdiagnosis: Approximately 1 month. Definitive Diagnosis: Pathological biopsy of bronchoscopically obtained specimens. Treatment: The patient had a prior history of left upper lobectomy with bronchoplasty. Pathological examination of the surgical specimen confirmed Squamous Cell Carcinoma. During the second cycle of adjuvant chemotherapy, the patient developed an irritating cough, prompting multiple subsequent bronchoscopies with cryotherapy. Concurrent anti-infective therapy was maintained for over 1. 5 years. Therapeutic Agents: Piperacillin-tazobactam, penicillin G, Amoxicillin. Treatment Duration: More than 1. 5 years. Therapeutic Outcome: Cured.

**Figure 12:**
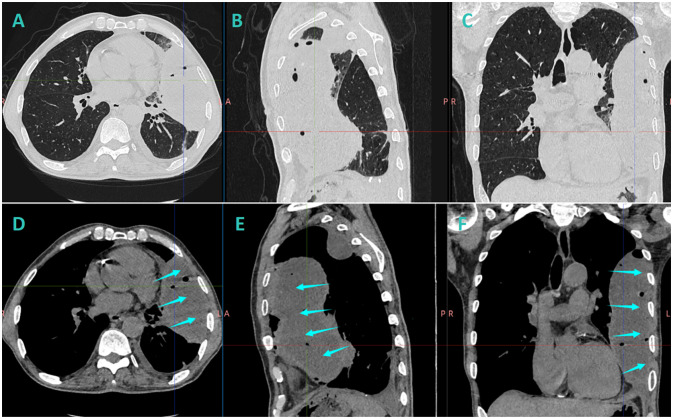
Classification of chest CT findings: type V: occult manifestation type. Images **(A-C)** (lung window, WW: 1500 HU; WL: -400 HU) **(D-F)** (mediastinal window, WW: 350 HU; WL: 50 HU): Clinical History: A 69-year-old man presented with cough, expectoration, and hemoptysis for 20 days. Chest CT Findings: Extensive atelectasis was present in the left lower lobe, associated with a mass-like consolidation of heterogeneous attenuation. Pericardial thickening and pericardial effusion were noted. A fluid-attenuation shadow [blue arrow in **(D–F)**] was observed in the left pleural cavity. Initial Misdiagnosis: Lung cancer. Duration of Misdiagnosis: Approximately 2 weeks. Definitive Diagnosis: Pathological biopsy of bronchoscopically obtained specimens. Treatment: During hospitalization, closed thoracostomy and bronchial artery embolization (BAE) were performed, with concomitant anti-infective therapy. Symptoms significantly improved postoperatively. Following discharge, anti-infective therapy was continued for nearly one year. Therapeutic Agents: Levofloxacin, Penicillin G, Amoxicillin. Treatment Duration: Over 1 year. Therapeutic Outcome: Cured.

**Figure 13:**
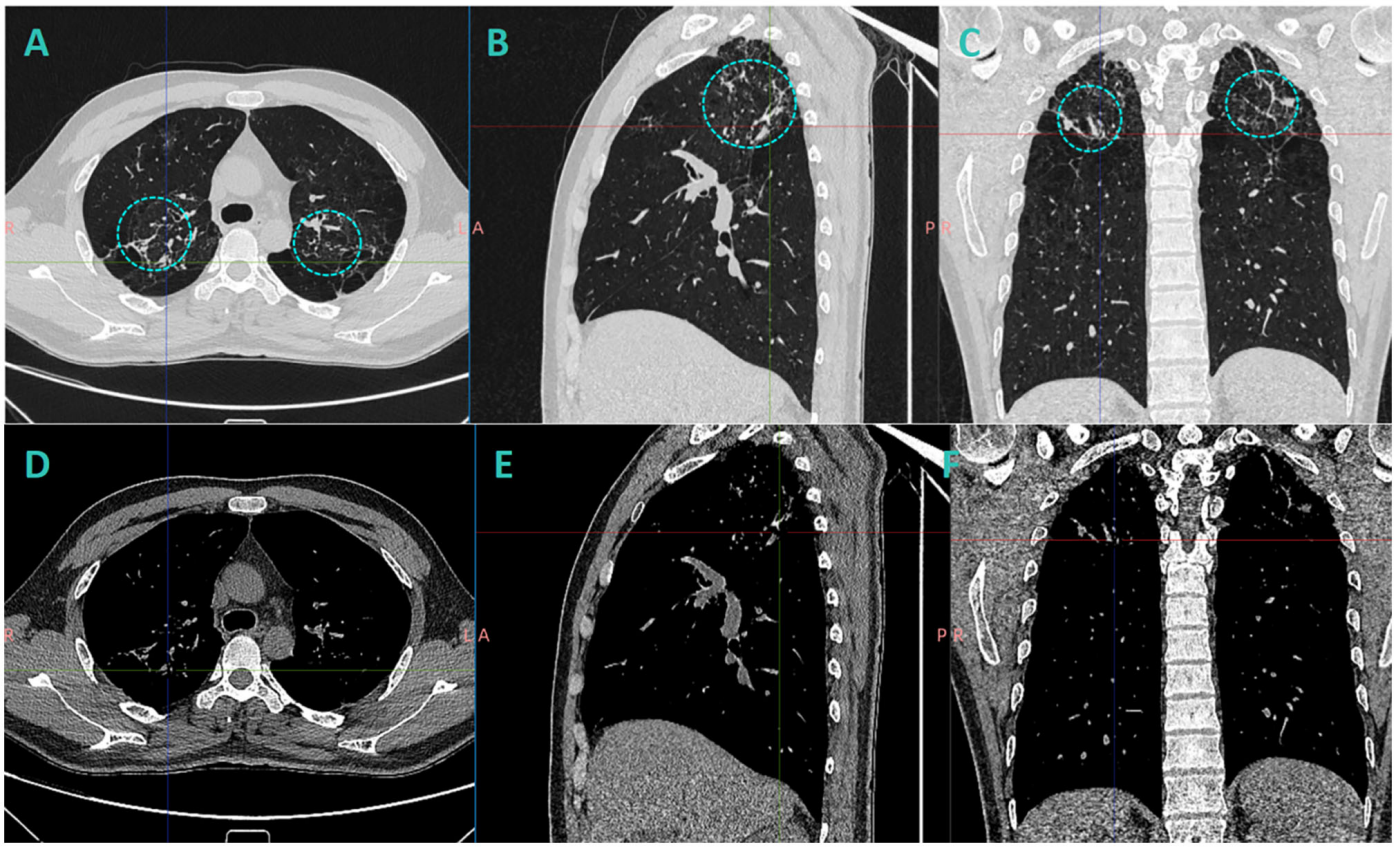
Classification of chest CT findings: type V: occult manifestation type. Images **(A-C)** (lung window, WW: 1500 HU; WL: -400 HU)| **(D-F)** (mediastinal window, WW: 350 HU; WL: 50 HU): Clinical History: A 29-year-old man presented with intermittent cough and expectoration for 6 months. Chest CT Findings:Multiple clustered nodules, patchy opacities, and linear opacities were seen in both lungs, with scattered calcifications [blue circles in **(A–C)**]. Focal pleural thickening with adhesion was noted bilaterally. Initial Misdiagnosis: Recurrent pulmonary tuberculosis. Duration of Misdiagnosis: 6 months. Definitive Diagnosis: Pathological examination of expectorated material. Treatment: The patient had a history of pulmonary tuberculosis (Mycobacterium tuberculosis identified in sputum culture), which was successfully treated with anti-tuberculosis therapy. Following confirmed diagnosis of actinomycosis infection, intravenous benzylpenicillin therapy was initiated, transitioning to oral amoxicillin for maintenance therapy over 1 year. Therapeutic Agent: Penicillin G, Amoxicillin. Treatment Duration: Over 1 year. Therapeutic Outcome: Cured.

**Figure 14:**
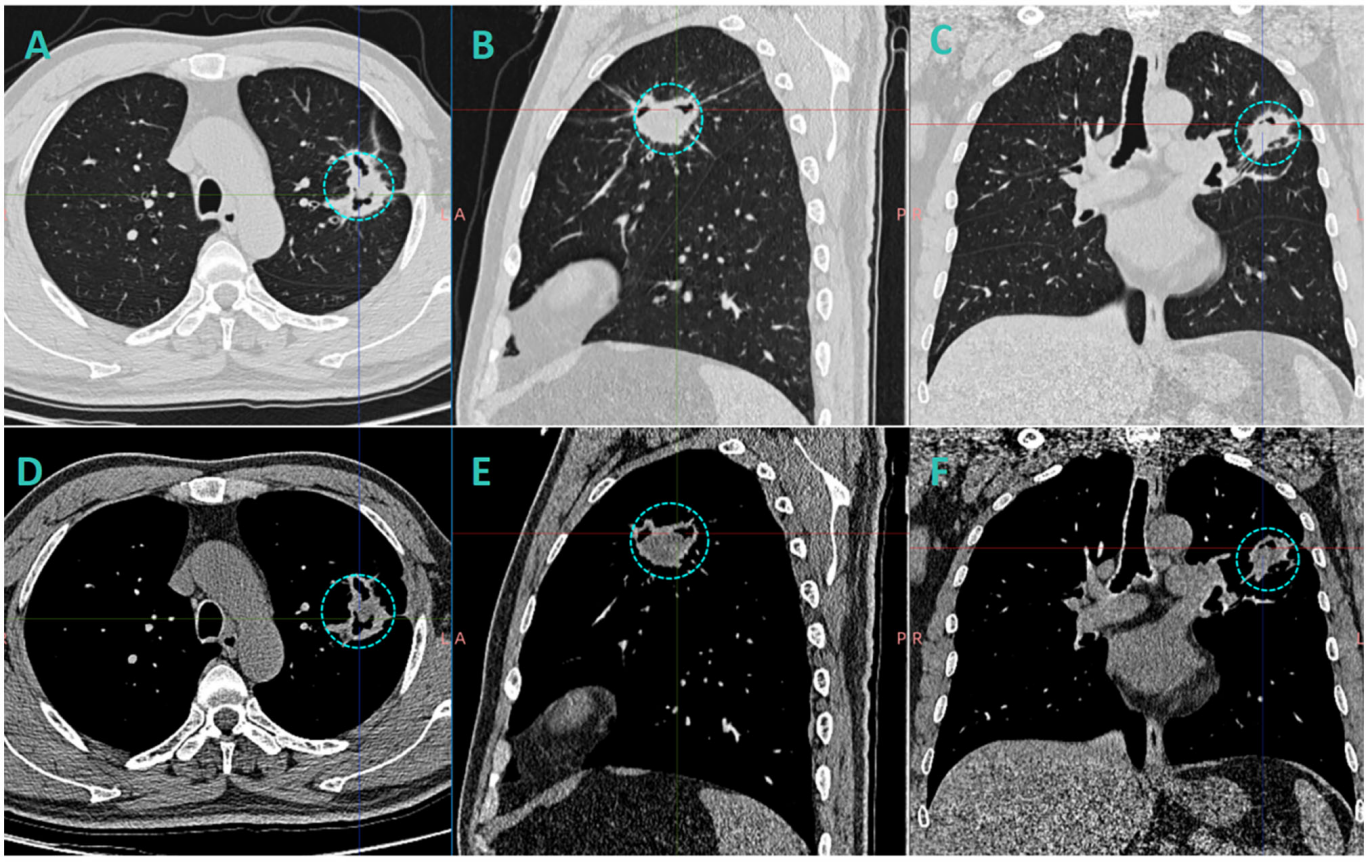
Classification of chest CT findings: type V: occult manifestation type. Images **(A-C)** (lung window, WW: 1500 HU; WL: -400 HU) | **(B-D)** (mediastinal window, WW: 350 HU; WL: 50 HU): Clinical History: A 50-year-old man presented with hemoptysis for over 1 year. Chest CT Findings: An irregular mass was visualized in the left upper lobe, demonstrating lobulated contours, spiculated margins, and pleural retraction. The lesion contained soft-tissue attenuation components and low-attenuation liquefactive necrosis [blue circles in **(A–F)**], with CT attenuation values ranging from -107. 00 HU to 119. 00 HU (mean: 33. 26 HU). Initial Misdiagnosis: Pulmonary tuberculosis. Duration of Misdiagnosis: Over 1 year. Definitive Diagnosis: Histopathology of surgical specimen. Treatment: Surgical intervention was performed, followed by postoperative intravenous cephalosporins for 2 weeks. No maintenance antibiotic therapy after discharge. Surgical Procedure: Left upper lobectomy. Postoperative Complications: None. Treatment Duration: Over 2 weeks. Therapeutic Outcome: Cured.

### Bronchoscopic findings and endoscopic interventions

In a subset of cases, bronchoscopic findings were minimal, demonstrating normal or near-normal lumina, or non-specific changes including edema, hyperemia, mucosal friability, and thickening. Characteristic secretions were observed in 28 cases (40. 6%): thin, milky-white purulent secretions or thick, cheese-like white purulent secretions. Additional findings included: grayish-white or white necrotic debris formation in 8 cases (11.6%); neoplastic growth causing luminal obstruction in 6 cases (8.7%); foreign body obstruction in 2 cases (2.9%); white patchy deposits in 3 cases (4.3%); white material at the bronchial stump in 1 case (1.4%); active hemorrhage in 2 cases (2. 9%); old blood clots in 2 cases (2.9%); and severe luminal stenosis in 1 case (1. 4%).

### Diagnostic confirmation

Definitive diagnosis was established in all 69 cases based on pathological examination, or metagenomic next-generation sequencing (mNGS) for species identification, or molecular biological methods. Confirmation was achieved using non-surgical specimens in 29 cases (42.0%), specifically: bronchoscopic biopsy in 24 cases (34.8%); computed tomography (CT)-guided percutaneous lung or pleural biopsy in 4 cases (5.8%); and expectorated specimen biopsy in 1 case (1.4%). Surgical resection specimens provided pathological confirmation in 40 cases (58.0%). Among these surgical cases, mNGS and molecular biological methods were subsequently performed on postoperative specimens for reconfirmation in 2 cases.

### Histopathological findings

All specimens underwent routine hematoxylin and eosin (H&E) staining. Special stains (e.g., Gomori methenamine silver, periodic acid-Schiff) were not performed. Gross examination revealed focal firm areas in 22 cases (31.9%), cystic cavities within lung parenchyma in 34 cases (49.3%), grayish-yellow necrotic debris in 24 cases (34.8%), and tan-to-gray necrotic coagulum in 20 cases (29.0%). Microscopic analysis demonstrated: Sulfur granules in 65 cases (94.2%), eosinophilic filamentous aggregates resembling Actinomyces in 36 cases (52.2%), basophilic filamentous aggregates resembling Actinomyces in 28 cases (40.6%), predominant neutrophilic infiltration in 16 cases (23.2%), lymph nodes exhibited reactive hyperplasia in 8 cases (11.6%). Final pathological diagnoses included: “Bronchogenic cyst with actinomycosis” in 3 cases (4.3%), “Pulmonary tuberculosis with aspergilloma and superimposed actinomycosis” in 1 case (1.4%).

### Treatment outcomes

Among the 69 patients, 40 underwent surgical intervention. Procedures included: lobectomy, combined lobectomy, lobectomy with segmentectomy, or pneumonectomy, performed via thoracotomy or video-assisted thoracoscopic surgery (VATS). Lymph node dissection was concurrently performed in select cases. All surgical patients received postoperative intravenous antibiotic therapy with β-lactams, cephalosporins, or fluoroquinolones; multidrug regimens were administered to a subset.

Surgical outcomes: 36 patients (90.0%) achieved cure. Complications occurred in 20 cases (50.0%), including pleural space infection, surgical site infection, and bronchopleural fistula. Two patients (5.0%) died postoperatively. Post-discharge management: 27 patients continued regular oral antibiotics (e.g., amoxicillin) for 2–16 months; 13 required no further anti-infective therapy.

Non-surgical treatment was administered to 29 patients, including 4 who underwent bronchial artery embolization to alleviate hemoptysis followed by continued anti-inflammatory therapy, and 13 who received treatments such as “foreign body removal, “ “cryotherapy, “ “laser therapy, “ or “tumor resection” before continuing anti-inflammatory therapy. Outcomes: 24 patients (82.8%) achieved cure. 1 experienced intermittent recurrence. 1 patient died of massive hemoptysis 41 days after discontinuing irregular antibiotic therapy. 4 demonstrated significant improvement.

The overall cure rate with surgical and non-surgical treatment was 87.0%, with a disease-specific mortality of 4.3%.

The follow-up period ranged from 14 to 150 months, with a median duration of 54 months (interquartile range [IQR] 36-75). During follow-up: 6 non-disease-related deaths occurred: 2 due to natural causes, 1 from coronary artery disease, 1 from heart failure, 2 from COVID-19. 5 patients were lost to follow-up (all cured at discharge): 2 at 24 months post-discharge, 2 at 12 months post-discharge, 1 at 18 months post-discharge.

Detailed therapeutic strategies, surgical intervention rates, non-operative management rates, treatment outcomes, operative complication rates, mortality counts, fatality rates, surgical cure rates, non-operative cure rates, and overall cure rates for each type are presented in **Table 2**. As shown in the table, all fatalities occurred in Type II, the most prevalent type. While Type II exhibited higher operative complication rates, it demonstrated superior surgical and overall cure rates. Type I showed lower operative complication rates compared to Type II, yet achieved the highest surgical and overall cure rates. Other types had limited surgical cases, rendering surgical metrics statistically insignificant.

**Table 2:** Proportions of CT imaging-based classifications, therapeutic approaches, treatment outcomes, and complications in pulmonary actinomycosis (N=69).

Category	Type I (n=6)	Type II (n=51)	Type III† (n=2)	Type IV (n=6)	Type V† (n=4)	*P* value
Surgical intervention						
Performed, n (%)	5 (83. 3)	32 (62. 7)	0 (0. 0)	1 (16. 7)	2 (50. 0)	0. 003**‡**
Complications, n/N (%)	2/5 (40. 0)	17/32 (53. 1)**§**	NA	1/1 (100. 0)	0/2 (0. 0)	0. 15
Cured, n/N (%)	5/5 (100. 0)	30/32 (93. 8)	NA	1/1 (100. 0)	2/2 (100. 0)	0. 58
Non-surgical management						
Performed, n (%)	1 (16. 7)	19 (37. 3)	2 (100. 0)	5 (83. 3)	2 (50. 0)	0. 21
Cured, n/N (%)	1/1 (100. 0)	14/19 (73. 7)	1/2 (50. 0)	4/5 (80. 0)	2/2 (100. 0)	0. 21
Overall outcomes						
Cured, n (%)	6 (100. 0)	44 (86. 3)	1 (50. 0)	5 (83. 3)	4 (100. 0)	0. 04**‡**
Mortality, n (%)	0 (0. 0)	3 (5. 9)	0 (0. 0)	0 (0. 0)	0 (0. 0)	0. 25

*1. P* < 0. 05 considered statistically significant (marked with**‡**).

2. NA = Not applicable (no cases in subgroup).

3. Type III excluded from surgical outcome comparisons due to null cases.

4. High complication risk suggests need for meticulous bronchial stump management and prolonged antibiotics (marked with **§**).

5. Insufficient subgroup sample size [n<5] for inferential statistics (marked with**†**).

## Discussion

This study systematically analyzed chest CT manifestations in 69 cases of pulmonary actinomycosis and proposes a novel five-type classification system: *Type I: Nodular infiltrative type. Type II: Airspace suspension type. Type III: Bilateral disseminated type. Type IV: Airway-originated type. Type V: Occult manifestation type.*

We further investigated the correlations between these CT patterns and therapeutic strategies/prognostic outcomes. The results demonstrate that CT-based classification significantly improves preoperative diagnostic accuracy, reduces misdiagnosis rates, and provides a foundation for personalized treatment. This system addresses a critical gap in current imaging research on pulmonary actinomycosis, potentially offering new evidence-based guidance for clinical decision-making.

Pulmonary actinomycosis is a rare infectious disease caused by Actinomyces species, characterized by localized suppuration and fibrogranulomatous inflammation with associated swelling (Wu and Qin, 2024). Clinical presentations often involve multiple organ systems, with non-specific symptoms, CT findings, and bronchoscopic features (Wang et al., 2020) that mimic pulmonary tuberculosis, lung abscess, bronchogenic carcinoma, fungal infections, and nocardiosis. In our cohort, only 6 cases (8.7%) were initially suspected upon admission, indicating substantial diagnostic challenges.

Cheon et al. previously categorized thoracic actinomycosis by anatomical involvement into parenchymal, airway (bronchiectatic/endobronchial subtypes), and extrapulmonary (mediastinal/chest wall) forms (Han et al., 2013). However, a CT imaging-based classification system remains undocumented. Our proposed classification system represents a refinement and radiological concretization of the anatomical classification proposed by Cheon et al., and establishes a link between imaging morphology and clinical outcomes (such as cure rates and postoperative complications), thereby providing a more detailed reference for preoperative planning.

Through comprehensive analysis of 69 patients over 21 years—incorporating clinical histories, lesion distribution, imaging features, clinical outcomes (particularly postoperative complications), bronchoscopic characteristics, and literature review—we classified CT manifestations into the following 5 types:

### Type I: nodular infiltrative type

Lesions are typically less than 3 cm in diameter, representing the early stage of pulmonary actinomycosis, often presenting as localized parenchymal infiltration. Specific manifestations include small, ill-defined nodules, linear opacities, microcavities, with or without small liquefactive necrotic foci. Infiltration of the bronchi can lead to localized bronchiectasis or stenosis, while involvement of the pulmonary interstitium results in thickening of the interlobular septa or fissures (Ibáñez-Nolla et al., 1993) (**Figure 6**). Although the disease extent is limited, hemoptysis may still occur, usually presenting as minor bleeding. Perilesional hazy shadows may be observed, correlating with aspiration pneumonia secondary to bleeding. This type closely mimics pulmonary tuberculosis or localized bronchiectasis, rendering differential diagnosis challenging and contributing to high misdiagnosis rates.

### Type II: airspace suspension type

If early-stage infection is not promptly treated with appropriate antibiotics, the nodules may gradually enlarge, accompanied by worsening bronchiectasis and increasingly prominent suppurative changes, ultimately evolving into the *Airspace suspension type*. Lesions are mostly larger than 3 cm, with lesions measuring 5–9 cm demonstrating the most characteristic features. Central suppurative necrosis forms hypodense areas accompanied by variably sized airspace/air-containing cavities (*Airspace suspension Sign*). The necrosis may be unifocal or multifocal, presenting as multicystic necrotic foci. Contrast-enhanced CT typically reveals no significant enhancement in these necrotic regions. Histopathological examination has confirmed the presence of actinomycotic colonies or sulfur granules within these areas.

Distinct from lung abscesses or pulmonary cysts, actinomycosis typically does not form a classic air-fluid level. The distribution of these cavities is independent of gravity; they are often suspended within the necrotic core rather than occupying the most superior position (*Airspace suspension Sign*). The underlying mechanism remains unclear but may involve local gas production from bacterial tissue destruction, with a vacuum state inside the cavity causing the gas to suspend within it, thus generally preventing the formation of a typical air-fluid level. The peripheral enhancing rim is composed of granulation tissue and fibrotic changes. With further progression, pulmonary parenchymal infection may extend across fissures to involve adjacent lobes, pleura, and the chest wall, potentially leading to abscess formation (**Figure 3**).

This is the most frequent clinical type, accounting for approximately 73. 9% of cases in this study, and its CT manifestations are also the most typical. Cheon et al. stated that the characteristic CT finding of pulmonary actinomycosis is chronic segmental airspace consolidation containing low-attenuation areas with peripheral enhancement and adjacent pleural thickening (Lin et al., 2019). Additionally, it may be accompanied by associated nonspecific manifestations such as pulmonary nodules, interlobular septal or interlobar fissure thickening, bronchiectasis, mediastinal lymph node enlargement, pleural thickening, pleural effusion, and empyema.

Although chest wall invasion or sinus tract formation has been historically described as a clinical characteristic of pulmonary actinomycosis, this feature is now rarely observed, likely due to improved disease recognition and timely antibiotic treatment. However, occasional cases are still reported (Ellebrecht et al., 2019; Boot et al., 2023). At our institution, a single case of actinomycosis was observed in a 33-year-old female patient who developed sinus tract formation with extrapleural extension. Computed tomography (CT) revealed lesion penetration through the intercostal muscles into the mammary gland, resulting in multiple abscess formations (**Figure 7**).

This type is most frequently misdiagnosed as bronchogenic carcinoma, pulmonary tuberculosis, or tuberculous empyema. According to Earle B. Kay, similar to tuberculosis, the actual extent of actinomycotic invasion is often significantly wider than the preoperative imaging suggests (Kay, 1946). The organism exhibits high invasiveness, often disregarding anatomical barriers, which may be attributed to the production of proteolytic enzymes facilitating spread from the lung to the pleura, mediastinum, and other structures, often resulting in trans-segmental and trans-lobar disease (**Figure 4**).

### Type III: bilateral disseminated type

**Figure 15:**
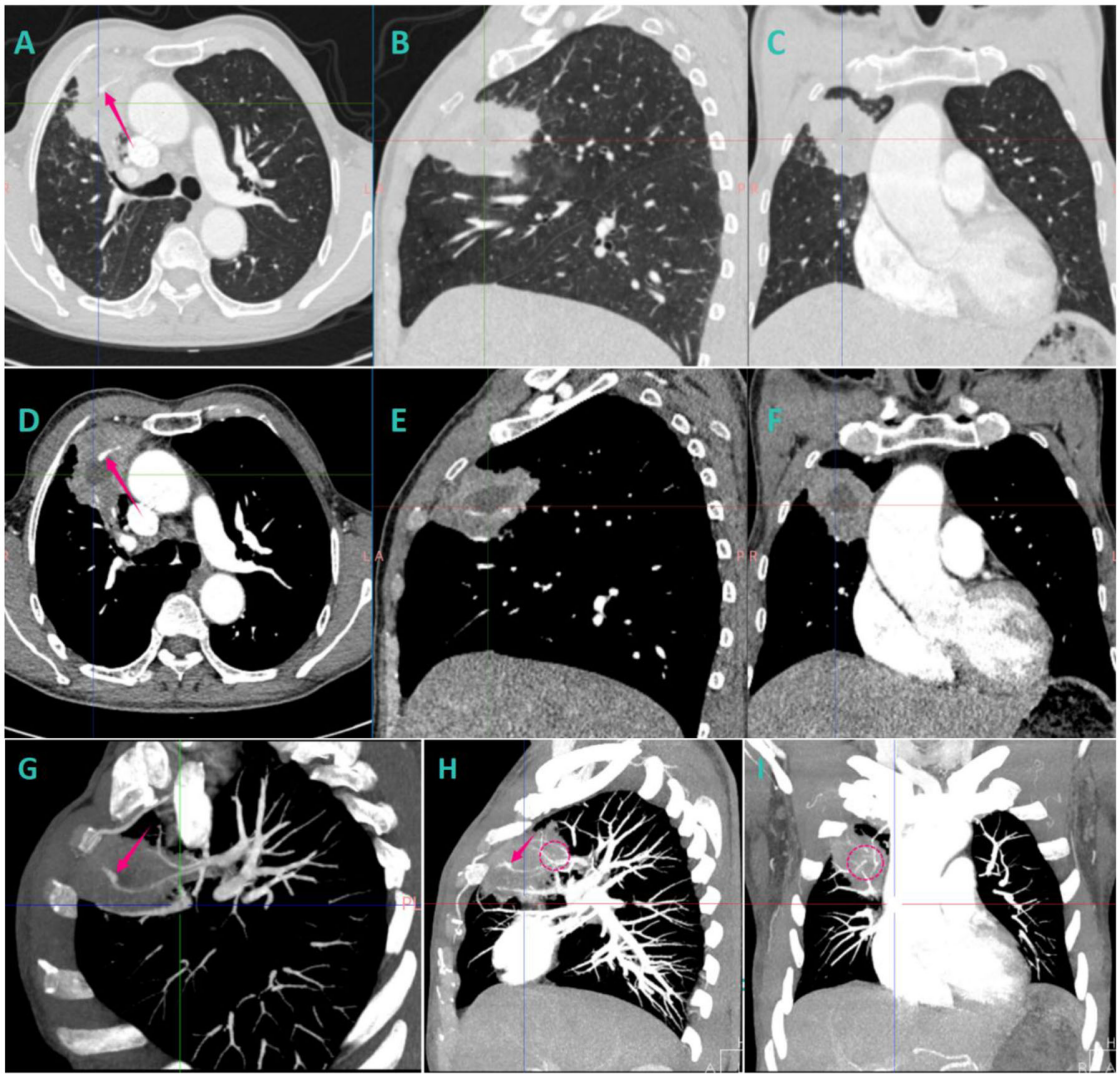
Vascular traversing sign and vascular destruction sign. Images **(A-C)** (lung window, WW: 1500 HU; WL: -400 HU) | **(D-F)** (mediastinal window, WW: 350 HU; WL: 50 HU) | **(G-I)** (angiography, WW: 400 HU; WL: 60 HU): Clinical History: A 66-year-old man presented with a one-week history of intermittent cough, expectoration, and hemoptysis. Chest CT Findings: The anterior segmental bronchus of the right upper lobe was occluded. An irregular mass, measuring approximately 54. 30 mm × 38. 00 mm, was identified in the anterior segment of the right upper lobe. The lesion exhibited heterogeneous attenuation with pronounced peripheral heterogeneous enhancement on post-contrast images (CT values: 27. 00 HU to 171. 00 HU; mean 76. 82 HU). Non-enhancing areas of liquefactive necrosis (CT values: -28. 00 HU to 71. 00 HU; mean 15. 91 HU) and the angiogram sign *(vascular traversing sign*) were observed within the mass [red circles in **(H, I)**]. A distal branch of the pulmonary artery demonstrated localized dilatation with aneurysmal formation *(vascular destruction sign)* [red arrows in **(A, D, G, H)**]. Initial Misdiagnosis: Lung cancer. Duration of Misdiagnosis: 1 week. Definitive Diagnosis: Histopathology of surgical specimen. Treatment: Surgical intervention was performed. Postoperative intravenous cephalosporin therapy was administered for anti-infective prophylaxis during the first week of hospitalization. Following discharge, oral amoxicillin maintenance therapy was continued for one year. Surgical Procedure: Right upper lobectomy. Postoperative Complications: Pulmonary infection and atelectasis of the remaining lung. Management of Complications: Antibiotic therapy and respiratory function exercises. Total Treatment Duration: Over 1 year. Therapeutic Outcome: Cured.

In rare instances, actinomycosis may manifest as disseminated bilateral pulmonary involvement, mimicking hematogenous miliary tuberculosis. This presentation is clinically uncommon, primarily documented in case reports (Nowicka et al., 2014; Xu and Shi, 2018). The lungs may be the sole affected organs or represent a component of systemic dissemination. Reports by Urszula Nowicka (Nowicka et al., 2014) and Xu, et al (Xu and Shi, 2018). describe similar cases. Additionally, untreated actinomycosis may progress to bilateral Airspace suspension lesions. For example, a 51-year-old woman in our cohort presented with a 4-month history of intermittent cough and more than 2 months of hemoptysis (poorly controlled) (**Figure 8**). CT-guided biopsy confirmed actinomycosis. Angiography of the bronchial artery, intercostal arteries, left internal thoracic artery, thoracoepigastric artery, and left gastric artery revealed tortuous and disorganized distal vasculature with associated vascular malformation networks and fistulous communications to the pulmonary artery (**Figure 15**). Subsequent transcatheter embolization resulted in symptomatic resolution.

This type requires the most careful differentiation from bilateral multifocal tuberculosis. The latter typically exhibits classic tree-in-bud signs, satellite lesions, or multiple cavities. On contrast-enhanced chest CT, centrally located liquefactive necrosis with peripheral ring enhancement may be observed within the lesions; however, it rarely demonstrates changes analogous to the relatively characteristic *Airspace suspension Sign* seen in pulmonary actinomycosis.

### Type IV: airway-originated type

This type has previously been termed endobronchial actinomycosis by some scholars (Sun et al., 2015; Posadas Blázquez et al., 2020). The primary focus resides within the bronchial lumen, with major risk factors including bronchial masses, bronchiectasis, bronchial foreign bodies, broncholithiasis, and endobronchial tuberculosis. Chest CT may demonstrate opaque foreign bodies with surrounding bronchial wall thickening and distal obstructive changes. Secondary findings such as consolidation, atelectasis, bronchiectasis, and pleural effusions may also be present. Calcified densities or foreign bodies are occasionally observed on chest CT or bronchoscopy, typically resulting from impacted broncholiths or foreign bodies that incite adjacent airway inflammation, organization, erosion, and distal pulmonary atelectasis with obstructive pneumonitis. CT reveals compromised bronchial patency and segmental or lobar atelectasis (**Figure 9**). Furthermore, studies indicate associations between endobronchial actinomycosis and endobronchial masses (**Figure 10**). While chest CT may fail to detect small endobronchial lesions, it can demonstrate bronchial stenosis or distal parenchymal consolidation/atelectasis (**Figure 10**). Erosion of calcified lymph nodes into airways may form broncholiths that subsequently trigger actinomycosis, often with prolonged clinical latency. Endobronchial actinomycosis secondary to broncholithiasis manifests on CT as proximal obstructing calcified bronchial nodules with distal obstructive pneumonia involving lobes or segments (Kim et al., 2005). These endobronchial presentations can mimic endobronchial tuberculosis or bronchogenic carcinoma (Afsin and Bacaksu, 2022). Symptoms are often subtle or absent, with many cases discovered incidentally during screening or unrelated investigations. Additionally, suture-induced actinomycosis may develop at pulmonary bronchial stumps, with parenchymal findings limited to nonspecific opacities, linear strands, and focal atelectasis (**Figure 11**). A prior retrospective study from our institution classified bronchoscopic manifestations into five patterns, providing a diagnostic reference for bronchogenic pulmonary actinomycosis (Zhang et al., 2021).

### Type V: occult manifestation type

This pattern frequently arises secondary to or concurrently with other pathologies, with the primary nidus often originating extraparenchymally. It is subclassified as follows:

**i.** Pleural effusion-dominant subtype.

This variant exhibits the greatest diagnostic challenge in distinguishing from tuberculous pleuritis, resulting in frequent misdiagnosis and mismanagement. Characteristic CT manifestations include: Free-flowing or loculated pleural effusions;Empyema. And secondary pulmonary alterations include: Compression atelectasis; Linear and patchy opacities (**Figure 12**).

Underlying pulmonary lesions may become detectable post-drainage and lung re-expansion. However, isolated pleural effusions without discernible parenchymal abnormalities have been documented (Seong et al., 2020). Actinomycosis frequently demonstrates invasion across anatomical barriers such as pulmonary septa, interlobar fissures, and pleural membranes. Consequently, associated findings often include interlobar pleural effusions, thickened pericardium, and pericardial effusion.

ii. Secondary/Comorbid subtype (Sarda et al., 2021).

Pulmonary actinomycosis may also occur secondary to or co-occur with other pulmonary pathologies, such as pulmonary tuberculosis (**Figure 13**), lung cancer (Kerget et al., 2024), lung abscess (Farrokh et al., 2014), and pulmonary cysts (**Figure 14**). This overlap in imaging features renders its pulmonary manifestations more subtle, increasing the likelihood of misdiagnosis or delayed diagnosis and thus posing greater challenges for clinical differential diagnosis.

Pulmonary actinomycosis is recognized as one of the most frequently misdiagnosed diseases. Minimizing diagnostic errors and missed diagnoses, while avoiding unnecessary surgical intervention, presents a significant challenge for clinicians. Drawing upon 21 years of cumulative clinical experience in diagnosing and managing pulmonary actinomycosis, we strongly recommend that lesions exhibiting the aforementioned imaging characteristics, particularly in patients demonstrating the typical *Airspace suspension type*, should be actively pursued with bronchoscopy or CT-guided transthoracic needle biopsy. To mitigate the risk of diagnostic failure due to insufficient tissue sampling, serial biopsies may be performed when necessary. Additionally, metagenomic next-generation sequencing (mNGS), microbiota analysis, molecular diagnostics (e.g, 16S rRNA PCR) can enhance diagnostic yield and reduce misdiagnosis rates. Currently, the mechanisms underlying hemoptysis in pulmonary actinomycosis and certain aspects of its imaging manifestations remain incompletely elucidated. Furthermore, no consensus has been established regarding optimal treatment regimens, surgical indications, or the management of postoperative complications.

The CT classification system proposed in this study is based on clinical research and retrospective analysis of a case series comprising 69 patients over a 21-year period. This cohort essentially encompasses the full spectrum of clinical characteristics observed in this pathology at our institution. According to our experience, different CT imaging types demonstrate some correlation with the selection of treatment modalities and the incidence of postoperative complications following surgical intervention:

For *Nodular infiltrative type* lesions located in the peripheral lung parenchyma, surgical options such as wedge resection of the lobe or anatomic segmentectomy are entirely viable. Postoperative complications, including pleural infection or surgical site infection, are virtually nonexistent following these procedures. However, when nodules are larger, more extensive, or situated in deeper parenchymal regions, lobectomy remains necessary (**Figure 6**).

The *Airspace suspension type* carries the highest risk of postoperative complications. Treatment strategy selection depends on the extent of disease involvement: Lesions confined to a single lobe or segment typically exhibit a low complication rate following complete resection. This observation is corroborated by the research of Chinese scholar SUN XF (Sun et al., 2015). However, lesions exhibiting extensive involvement, crossing lobar or segmental boundaries, or invading the interlobar fissure, chest wall, or pleura are associated with a significantly higher incidence of postoperative complications. Intraoperatively, meticulous exploration and complete lesion excision are imperative. When necessary, combined lobectomy and segmentectomy or multilobar resection should be performed. Reinforcement and coverage of the bronchial stump are essential to reduce the risk of bronchopleural fistula. Postoperatively, intensified or combination anti-inflammatory therapy is required. For penicillin monotherapy, the principles of “intensive and prolonged” therapy must be adhered to: intravenous penicillin G at high doses (18–24 million units daily) for 2–6 weeks, followed by oral penicillin V or amoxicillin for 6–12 months. Cases with extrathoracic penetration also demonstrate a higher propensity for postoperative complications, such as sinus tract infections and wound dehiscence.

For the *Bilateral disseminated type*, conservative medical management is recommended as the primary approach. Due to the extensive or diffuse nature of the disease in this subtype, resection of specific lesions is not feasible; however, these cases often respond favorably to anti-inflammatory therapy or localized interventions. As illustrated in **Figure 8**, one patient presented with recurrent hemoptysis. Following bronchial artery embolization for hemostasis, symptoms markedly improved. The patient continued outpatient antimicrobial therapy for nearly one year and has remained stable with no recurrence during follow-up. In another representative case of this subtype, hemoptysis did not recur following antimicrobial therapy alone.

For the *Airway-originated type*, outcomes are generally favorable if foreign bodies (**Figure 9**) or broncholiths can be successfully removed endoscopically, combined with aggressive antimicrobial therapy. Some patients achieve complete resolution after undergoing bronchoscopic mass resection (**Figure 10**) or serial cryotherapy sessions (**Figure 10**), followed by intensive antimicrobial treatment. When endoscopic management proves difficult or fails, surgical intervention is indicated and typically results in definitive cure.

For the *Occult manifestation type*, initial management consists of pleural fluid drainage and antimicrobial therapy, with a deferred decision regarding surgical intervention. In this study, two patients with this type achieved complete resolution following conservative management (**Figures 12**, **13**). The patient depicted in **Figure 13** demonstrated significant symptomatic improvement with antimicrobial therapy alone, subsequently achieving complete resolution after intensified antimicrobial treatment. The patient shown in **Figure 12** underwent tube thoracostomy and bronchial artery embolization followed by intensified antimicrobial therapy, resulting in complete resolution. An additional two patients also achieved complete resolution following surgical resection and postoperative antimicrobial therapy; one is illustrated in **Figure 14**, and the other had disease secondary to an aspergilloma.

Notably, research by Professor So Ri Kim from South Korea indicates that the most common clinical manifestation of this disease is hemoptysis, occurring in approximately 64.9% of cases. In our study, hemoptysis was reported as a chief complaint in 51 patients (73.9%). Among the 42 patients who underwent contrast-enhanced chest CT, 30 exhibited vascular changes within the lesions, including traversing, displacement, encasement, and traction of vessels, with distal vascular dilatation observed in some instances (**Supplementary Figure 1**). The precise mechanism underlying hemoptysis in pulmonary actinomycosis remains incompletely understood. We postulate that Actinomyces infection may induce vascular tortuosity, deformation, formation of abnormal vascular networks, collateral vessels, and vascular fistulae. Concurrently, erosion, destruction, dilatation, and rupture of vessels caused by the infection may contribute to hemoptysis. This hypothesis is supported by direct visualization of active bleeding from corresponding vessels during interventional angiography and embolization procedures in a subset of cases within our cohort (**Supplementary Figure 1**). Previous reports also corroborate this view. These characteristic chest CT findings may provide valuable insights for elucidating the mechanisms of hemoptysis in this disease.

## Conclusions

In conclusion, this study introduces and preliminarily validates the first CT-based classification system for pulmonary actinomycosis, which may improve preoperative diagnostic accuracy, guide type-specific therapeutic strategies, and reduce unnecessary surgical interventions (**Supplementary Figure 2**).

Statistically significant differences were observed in surgical requirements across the types (*P* = 0. 003), with marked variation in the necessity for surgical intervention: Type II exhibited the highest surgical rate (62. 7%) and a cure rate of 93. 8%; surgery is recommended as the primary approach (particularly for lesions >3 cm or those involving the chest wall). Type IV had the lowest surgical rate (16.7%), with a non-surgical cure rate of 80% (4/5); bronchoscopic intervention combined with antibiotics is recommended as the first-line treatment (to avoid unnecessary surgery). A cautionary note for Type IV (1 surgical case resulted in 100% complications) warrants careful assessment of surgical indications, with priority given to attempting endoscopic therapy. Type III was the only type with no surgical intervention (0% surgical rate). Its conservative treatment cure rate was only 50%, significantly lower than other types (overall cure rate comparison *P* = 0. 04) and representing the poorest treatment outcome; non-surgical management is mandatory (intensified anti-infective therapy combined with supportive care).

No significant differences in surgical complication rates were observed across types: Complication rates showed no statistical significance between groups (*P* = 0. 15). However, the surgical complication risk requires caution: Type II demonstrated the highest complication rate (53. 1%), clinically indicating the need for enhanced intraoperative and perioperative management (e. g., meticulous bronchial stump management, intensified anti-infective therapy) and prolonged postoperative antibiotic therapy for more than 12 months. The 100% complication rate in Type IV (n=1 surgical case) necessitates cautious interpretation due to the small sample size.

Regarding treatment outcomes, both Type I and Type V achieved 100% cure rates regardless of surgical or conservative management, representing the subtypes with the most favorable prognosis, allowing for individualized treatment selection. Type I showed a 100% surgical cure rate (5/5) with a complication rate of 40%; early minimally invasive surgery (wedge/segment resection) is recommended. Type V tends to be managed successfully with drainage combined with antibiotic therapy.

Although the overall mortality rate in this cohort was low (4. 3%), all three fatal cases were exclusively concentrated in Type II (3 cases, 5. 9% of this type). No mortality was observed in other imaging types (Type I, III, IV, V), a finding warranting clinical attention. Although statistical significance was not reached (*P* = 0. 25, potentially attributable to limited sample size, confounding factors, or the rarity of the mortality outcome), this finding aligns with the high complication rate (53. 1%) associated with surgical intervention in this type. The aggressive features of Type II lesions—characterized by interlobar infiltration and a propensity for bronchopleural fistula formation—may underlie this elevated risk. Therefore, we emphasize the necessity to optimize perioperative management for Type II patients, including meticulous bronchial stump reinforcement and prolonged antibiotic courses (>12 months), to mitigate potential mortality risk. This observation further underscores the clinical utility of this CT-based typing system in identifying high-risk subgroups and guiding targeted interventions.

### Limitations

This study has several limitations inherent to its single-center retrospective case analysis design. For instance, indications for surgical versus conservative management may exhibit interindividual variations. Additionally, the relatively short follow-up period has precluded sufficient assessment of associations between CT types and long-term outcomes, such as recurrence and survival rates. Furthermore, only two cases underwent comprehensive analysis incorporating metagenomic next-generation sequencing (mNGS), microbiota analysis, molecular diagnostics (e.g., 16S rRNA PCR), potentially affecting diagnostic specificity and accuracy. Future multicenter prospective cohort studies utilizing standardized classification systems and molecular pathological evidence are warranted to validate our findings. For Types III and IV with minimal representation, our results should be considered exploratory. However, they provide critical baseline data that will be incorporated into our planned, multi-center validation study, which specifically aims to enrich these subgroups.

To validate the external applicability of this classification system, a multicenter prospective validation study is currently being implemented. While Types III and IV were represented by smaller cohorts in this initial derivation study, their distinct clinical and radiological profiles have been clearly elucidated, providing a crucial foundation for our ongoing, prospective multi-center validation study aimed at specifically enriching these rare types. Furthermore, algorithm-based AI-assisted image analysis may serve as a primary tool to enhance diagnostic consistency in subsequent validations.

## Data Availability

The original contributions presented in the study are included in the article/**Supplementary Material**. Further inquiries can be directed to the corresponding author.

## Supplementary material

Supplementary Figure 1 Vascular destruction sign, abnormal vascular network, and arteriovenous fistula (same patient as in **Figure 8**). Angiographic findings: The distal branches of the intercostal artery (green arrow in **(A)**), thoracoepigastric artery (green arrow in **(B)**), left internal thoracic artery (LITA) (green arrow in **(C)**), and left inferior phrenic artery (green arrow in **(D)**) were tortuous and disorganized, with abnormal vascular networks observed (*vascular destruction sign, abnormal vascular network*) (circles in **(A–D)**). Additionally, Left thoracoepigastric artery to left pulmonary artery fistula (red circle in **(B)**) and left inferior phrenic artery to left pulmonary artery fistula (red circle in **(D)**) was identified (*arteriovenous fistula*).

Supplementary Figure 2 CT-Based Classification and therapeutic strategies in Pulmonary Actinomycosis. Clinically indicating the need for enhanced intraoperative and perioperative management (e. g., meticulous bronchial stump management, intensified anti-infective therapy) and prolonged postoperative antibiotic therapy for more than 12 months (marked with*).
