# Supplementary figures and images for: The first CT-based classification system for pulmonary actinomycosis: correlating imaging patterns with therapeutic strategies and prognosis in a highly misdiagnosed disease
Source: Front Cell Infect Microbiol. 2026 Jul 2;16:1836090. doi: 10.3389/fcimb.2026.1836090 (PMC13372579; doi:10.3389/fcimb.2026.1836090)

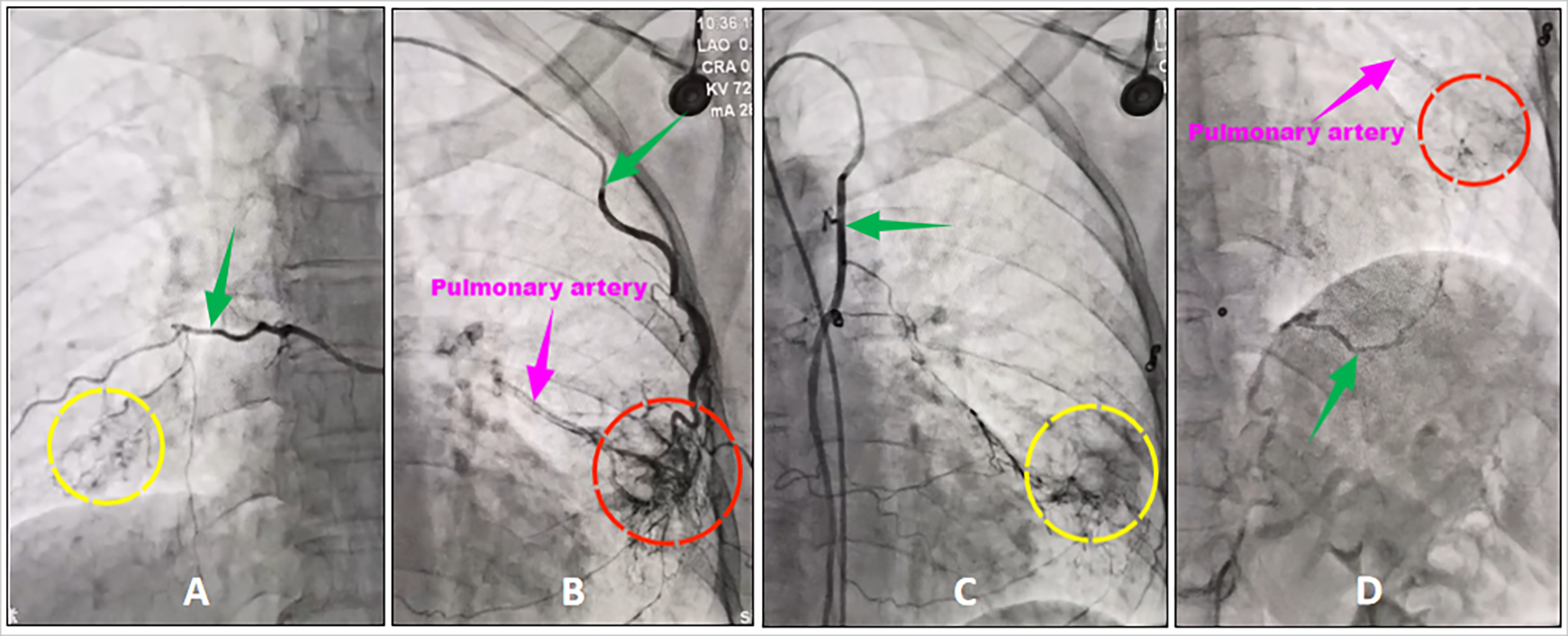

Supplement: Supplementary Figure 1 — Vascular destruction sign, abnormal vascular network, and arteriovenous fistula (same patient as in Figure 8). Angiographic findings: The distal branches of the intercostal artery (green arrow in (A)), thoracoepigastric artery (green arrow in (B)), left internal thoracic artery (LITA) (green arrow in (C)), and left inferior phrenic artery (green arrow in (D)) were tortuous and disorganized, with abnormal vascular networks observed (vascular destruction sign, abnormal vascular network) (circles in (A–D)). Additionally, Left thoracoepigastric artery to left pulmonary artery fistula (red circle in (B)) and left inferior phrenic artery to left pulmonary artery fistula (red circle in (D)) was identified (arteriovenous fistula). [file Image1.tif]

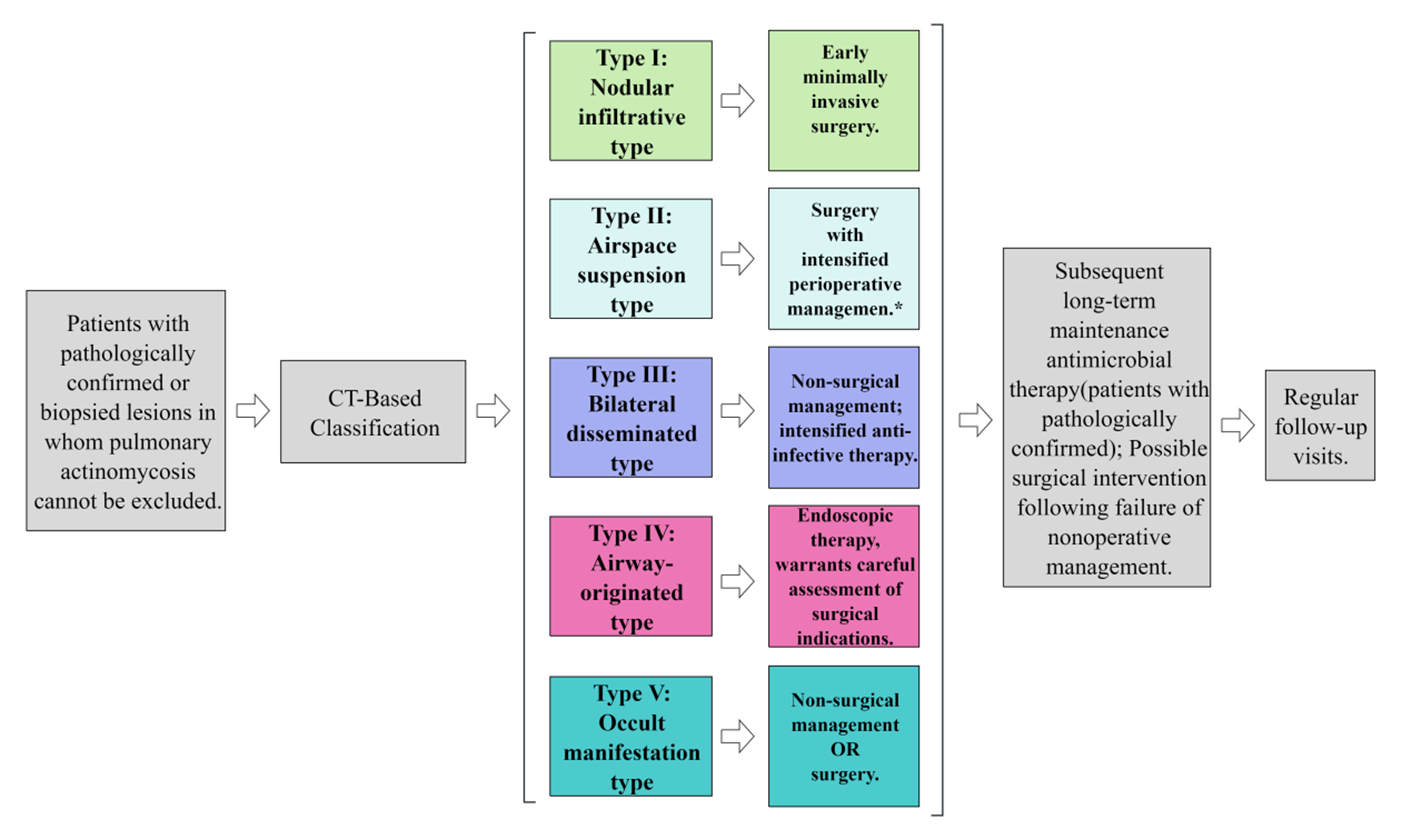

Supplement: Supplementary Figure 2 — CT-Based Classification and therapeutic strategies in Pulmonary Actinomycosis. Clinically indicating the need for enhanced intraoperative and perioperative management (e. g., meticulous bronchial stump management, intensified anti-infective therapy) and prolonged postoperative antibiotic therapy for more than 12 months (marked with*). [file Image2.tif]
